# A complete and dynamic tree of birds

**DOI:** 10.1073/pnas.2409658122

**Published:** 2025-04-29

**Authors:** Emily Jane McTavish, Jeff A. Gerbracht, Mark T. Holder, Marshall J. Iliff, Denis Lepage, Pamela C. Rasmussen, Benjamin D. Redelings, Luna L. Sánchez Reyes, Eliot T. Miller

**Affiliations:** ^a^Department of Life and Environmental Sciences, School of Natural Sciences, Merced, CA 95343; ^b^Cornell Lab of Ornithology, Cornell University, Ithaca, NY 14850; ^c^Department of Ecology and Evolutionary Biology, and the Biodiversity Institute, University of Kansas, Lawrence, KS 66045; ^d^Birds Canada, Port Rowan N0E 1M0, ON, Canada; ^e^American Bird Conservancy, The Plains, VA 20198

**Keywords:** phylogeny, open data, evolution, taxonomy

## Abstract

We present a complete, time-scaled, evolutionary tree of the world’s bird species. This tree unites phylogenetic estimates for 9,239 species from 262 studies published between 1990 and 2024, using the Open Tree synthesis algorithm. The remaining species are placed in the tree based on curated taxonomic information. The tips of this complete tree are aligned to the species in the Clements Taxonomy used by eBird and other resources, and cross-mapped to other taxonomic systems including the Open Tree of Life (Open Tree), National Center for Biotechnology Information, and Global Biodiversity Information Facility. The total number of named bird species varies between 10,824 and 11,017 across the taxonomy versions we applied (v2021, v2022, and v2023). We share complete trees for each taxonomy version. The procedure, software, and data stores we used to generate this tree are public and reproducible. The tree presented here is Aves 1.3 and can be easily updated with new phylogenetic information as new estimates are published. We demonstrate the types of large-scale analyses this data resource enables by linking geographic data with the phylogeny to calculate the regional phylogenetic diversity of birds across the world. We will release updated versions of the phylogenetic synthesis and taxonomic translation tables annually. The procedure we describe here can be applied to developing complete phylogenetic estimates for any taxonomic group of interest.

## Significance

Birds are charismatic—well loved, and highly studied. Many new phylogenies elucidating avian evolutionary relationships are published every year. We have united phylogenetic estimates from hundreds of studies to create a complete evolutionary tree of all birds. While a variety of resources aggregate huge collections of trait, behavior, and location data for birds, previously the barriers to linking data between these data resources and bird evolutionary history have limited the opportunities to do exciting large-scale analyses. We have bridged that gap and developed a system that allows us to easily update our understanding of bird evolution as new estimates are generated. We share a workflow and the software needed to create a complete evolutionary tree for any group.

Advances in data availability make it increasingly possible to address large-scale biological questions across the diversity of life. More researchers and project teams are providing open access to their primary data, which has accelerated the progress of integrative research. However, to build on this momentum, it is necessary to not only make data available but also to make it easy to dynamically apply those data to new research questions. The tree of life provides a comprehensive, biologically relevant means to link datasets and cohesively analyze data from multiple sources. Historically, limitations in access to evolutionary trees have resulted in a gap between the novel inferences of relationships between species generated by systematists, and the analyses researchers want to perform using the tree of life (1, 2). Only recently has it been possible to generate homologous data and estimate phylogenies for thousands of taxa, and even these very large phylogenies rarely have complete taxonomic representation. These data availability issues mean that large-scale estimates of biodiversity have often relied on outdated phylogenetic and taxonomic relationships, rather than cutting-edge evolutionary inferences (3).

The Open Tree of Life project (Open Tree, hereafter) bridges that gap by unifying phylogenetic estimates of relationships for taxa across the entire tree of life (1, 4). Open Tree provides a framework for linking names, metadata, and phylogenetic information for any set of taxa. Since the publication of the first draft tree in 2015 (4), Open Tree has developed into a robust biodiversity informatics resource. Here, we apply the synthetic tree building and taxonomy tools developed by Open Tree to provide a complete and updatable, time-scaled, tree of all bird species.

This project not only provides a complete evolutionary tree of all bird species, it also provides a framework for maintaining an accurate and readily available estimate of bird relationships, even as new species are discovered, and prior relationships and taxonomy are revised. The input data curation and the published data products all follow FAIR principles for data stewardship—Findability, Accessibility, Interoperability, and Reuse of digital assets (5). All the input phylogenies are available and have extensive curated metadata (6). The phylogenies in the data store are searchable both by their publication information, and by the actual phylogenetic and taxonomic information contained within. We developed and published taxonomic translation tables along with the tree that make it easy to link this tree with large existing bird datasets, including AVONET (7) trait data and eBird distribution data (8), among many others.

Our phylogenetic synthesis method seeks to summarize and represent the collection of published input trees in a clear and transparent fashion. In pursuit of this goal, each edge of our synthetic tree is annotated with the set of input trees that support and conflict with it, and each edge corresponds directly to an edge in at least one of the input trees. Most input trees do not have overlapping leaf sets, and we focus on combining published phylogenetic trees to achieve information for as many taxa as possible. While some supertree methods treat input trees as proxies for data matrices (9) and attempt to infer the true tree from these data (10, 11), our approach is not intended to, and cannot, produce a phylogeny estimate that is more accurate than the inputs (12). Further detail on the goals of our synthesis tree pipeline can be found in ref. 12.

The synthesis tree is updatable as new data become available. We version the tree, and the provenance information for each version includes a static record of the set of inputs and rankings used to generate each tree. The synthesis version described here is tagged as “Aves 1.3,” and each subsequent update will receive its own tagged version number. New versions of the tree will be added to the data store available on GitHub and published with a DOI on Zenodo. All data and code are publicly available, and the procedure to generate the tree is fully reproducible. This synthesis framework can also be applied to developing and maintaining a complete dynamic phylogenetic estimate for any taxonomic group of interest.

## Results.

**Fig. 1.**
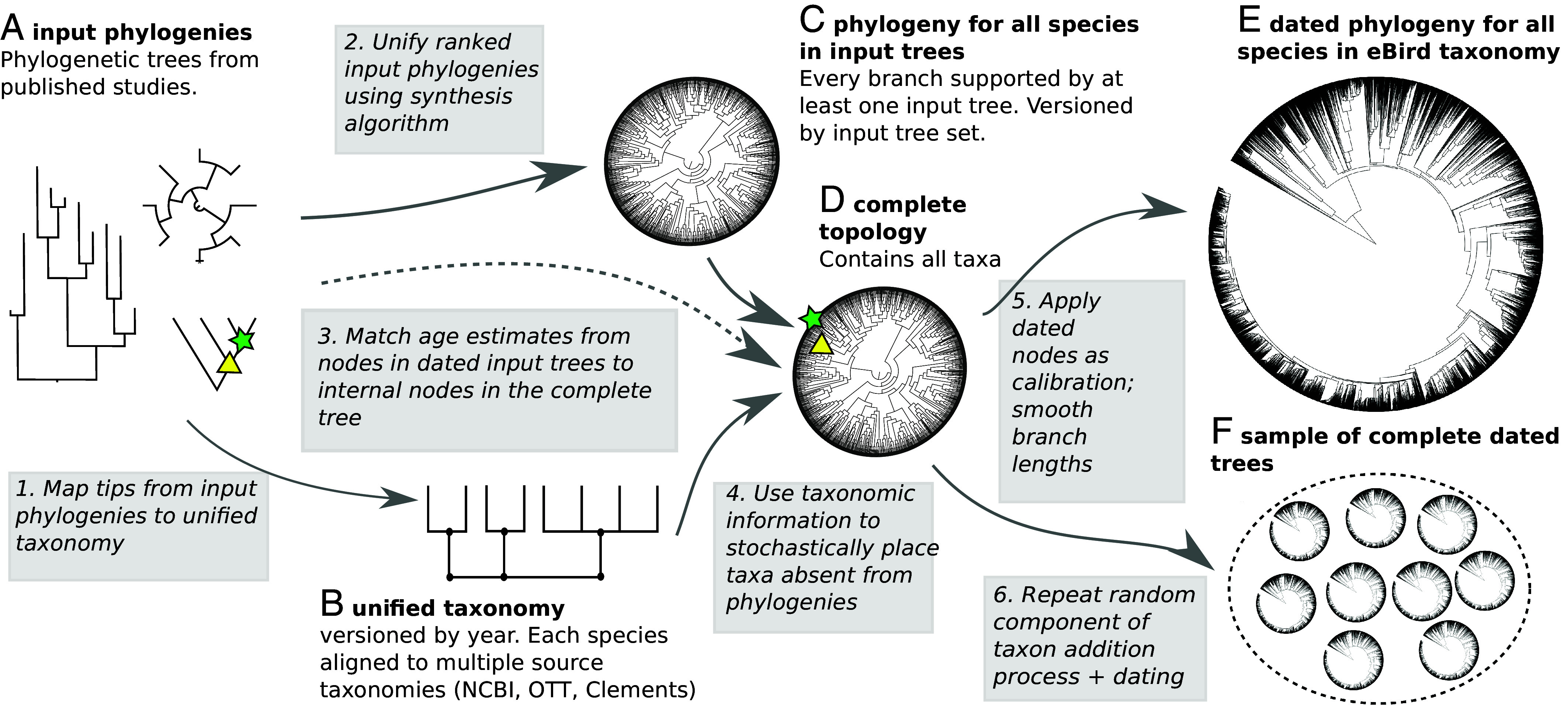
The phylogenetic synthesis workflow. Data products are labeled with letters (*A*–*F*) and are shared in the data repositories. Analysis steps are labeled with numbers (1 to 6) and rely on open source code.

**Fig. 2.**
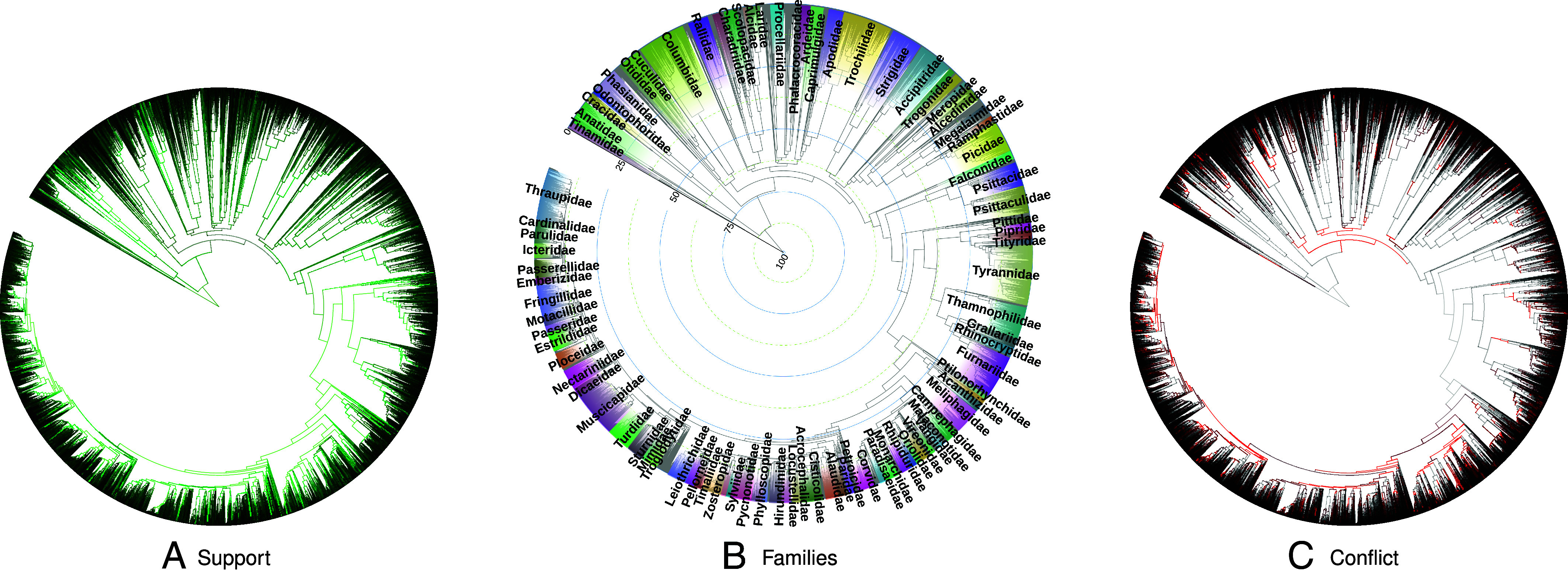
The complete tree labeled with (*A*) number of trees in support of each branch (grey—no supporting studies; dull green—1 to 19 supporting studies, brightest green—20 or more supporting studies). (*B*) Bird families with 25 or more members in the tree (with some exceptions due to space), and age estimates in millions of years ago (Mya). (*C*) Number of trees in conflict with each branch (grey—no conflicting studies, dull red—1 to 4 conflicting studies, brightest red—5 or more conflicting studies).

We unified 281 input phylogenies into a phylogenetic synthesis for 9,239 species across all birds (Figs. 1 and 2). We used curated taxonomic information to add in taxa which were absent from phylogenetic estimates to generate complete trees with all species present in the 2021, 2022, and 2023 Clements taxonomy versions (Fig. 1 and Table 1). The provenance of each branch can be traced back to the input studies which support that relationship (Fig. 2*A*). By using unique taxon identifiers associated with each taxonomy, tip labels on these species-level trees can be easily translated from Clements taxonomy to several other taxonomies.

### Data integration.

Although evolutionary data from published papers often are not readily available (see refs. 1 and 13), when they are, they can be shared in a variety of formats. Nearly all tree files require human curation to map tip labels to shared taxonomic identifiers and to ensure correct rooting and metadata collection. We curated 281 individual trees from 262 published studies and included them in the Open Tree phylesystem datastore (6), (Fig. 1). These trees comprise a total of 44,117 individual tips. We developed a taxonomic crosswalk table which contains unique identifiers from the Open Tree taxonomy (OTT v3.6) (14) for all 10,824 species in the Clements 2021 taxonomy, and most species in the 2022 (10,746/10,906; 99%) and 2023 (10,765/11,017; 98%) Clements taxonomy (15) (Table 1). We were able to match 35,534 (86%) of the input study tips to species in the Clements taxonomy via identifiers in OTT. We used Avibase taxon concepts associated with species in the Clements taxonomy to link identifiers for taxa whose names changed across years (16).

We had phylogenetic information, as defined by a species appearing in at least one input tree, for 9,239 of the 10,824 species in the Clements 2021 taxonomy [2022: 9,207/10,906; 2023 9,189/11,017 (Table 1)]. Some input tree labels could not be matched to some versions of the taxonomy, resulting in small differences in the counts of species for which we have phylogenetic information across taxon-years. New phylogenies and corrected name mappings can be contributed to the data store by any user at any time (6).

### Phylogenetic synthesis.

All internal nodes in the phylogeny-only tree are supported by at least one input phylogeny (Fig. 1*C*). We publish with the synthesis tree an annotation file tracing the provenance of every branch in the synthetic tree, including what input study and tree supports that branch’s inclusion in the final tree, what trees have branches that align with that branch but do not directly support that branch, and what trees contain relationships that conflict with that branch in the tree (12).

The majority of branches in the tree were supported by one to five phylogenies, although some nodes in the tree are supported by up to 24 input phylogenies (Fig. 2*A*). In a testament to the history of change in the estimated phylogenetic relationship of Aves over time, 3,781 branches (around 34%) conflict with at least one study (Fig. 2*C*). While in most cases of conflict there are one or two input studies that conflict with the resolution at a branch, there are 20 branches with 10 to 15 studies that show an alternate resolution for that relationship. As the synthesis algorithm is a greedy algorithm that prefers more recent inferences over older ones, it is possible to have to branches WHERE more studies conflict with a branch than support it. We observe this pattern in 14% of branches. These persistent conflicts may result from different inferences for trees using different data types (17).

**Table 1.** Species count and phylogenetic information across trees aligned to taxonomy versions

Clements taxonomy year	Total species count	Species in OTT	Species with phylogenetic information (%)
2021	10,824	10,824 (100%)	9,239 (85%)
2022	10,906	10,746 (99%)	9,207 (84%)
2023	11,017	10,807 (98%)	9,189 (83%)

To create a complete tree from the phylogenetic synthesis tree, we used a curated taxon addition step. To add taxa that we did not have phylogenetic information for we used the R package “addTaxa” (18) (Fig. 1 step 4). Taxa without phylogenetic information are added to the tree using curated taxonomic information files. These taxon information files provide a constrained region of the tree that experts believe the taxon should be placed in, using multiple data sources including Birds of the World (15). These files are versioned, and readily accessible and updatable. These taxa are then added stochastically based on these constraints. We sampled 100 random taxon addition trees per taxonomy year and, after dating the trees, summarized these sampled trees into a single maximum clade credibility tree. This tree summarizes the relationships into a single complete tree including all taxa. This summary tree includes CIs on node dates. We used this process to generate a complete tree for each of the 2021, 2022, and 2023 versions of the Clements taxonomy.

### Concordance and conflict.

In regions of the tree where there is conflict among studies, the choice of rankings for phylogenies affects the final tree. While we used chronological order as a ranking, with the most recent tree in the collection ranked most highly, there are arguments for alternative ranking schemes, such as based on inference method or data quality. The algorithm works as a reverse veto method—once a branch has been added, it will be present in the final tree, even if older studies contradict that relationship. To explore how ranking choices affect topology, we compared four alternate syntheses where different major Aves phylogenies were ranked highest. These phylogenies were Stiller et al. (363 species) (19) (this is the default under the chronological ranking in Aves 1.3), Wu et al. (124 species) (20), Prum et al. (198 species) (21), and Jetz et al. (6,647 species) (22), a tree that has been widely used for over a decade for ornithological studies (e.g. ref. 23). The published tree from Jetz et al. (22) contained 9,993 tips, but for this analysis, we used only the subset of those tips which were informed by genetic data (66%).

The full phylogenetic synthesis tree for each of these alternate rankings is shared in the data store. The symmetric differences (Robinson-Foulds distances) in branches between each of those trees and the Aves 1.3 tree were 176 [Wu et al. (20)], 170 [Prum et al. (21)], and 5,126 [Jetz et al. (22)]. Whether the top ranked tree is Stiller et al. (19), Wu et al. (20), or Prum et al. (21), less than 1% of branches differ among final synthesis trees, whereas nearly 14% of branches differ when the Jetz tree, which contains many more taxa, is ranked first.

In our synthetic tree, we have phylogenetic information placing the relationships of 8,414 (84%) of the taxa in the Jetz et al. tree (22). There are 2,377 branches in our tree that capture relationships that directly conflict with the Jetz et al. tree (*SI Appendix*, Fig. S2), and there are phylogenetic relationships for 2,451 taxa in this tree which were not present in the Jetz et al. tree or in any other previous single study. The symmetric difference when the Jetz et al. tree is ranked first is higher than the number of direct conflicts, as the rank order affects what branches from subsequent trees can be added to the synthesis estimate.

While the relationships in the Aves 1.3 phylogenetic synthesis tree remain essentially the same across all three taxonomy versions, there are some changes due to lumping or splitting of taxa, taxon name matching changes and pruning the tree to the species level (Table 1).

**Fig. 3.**
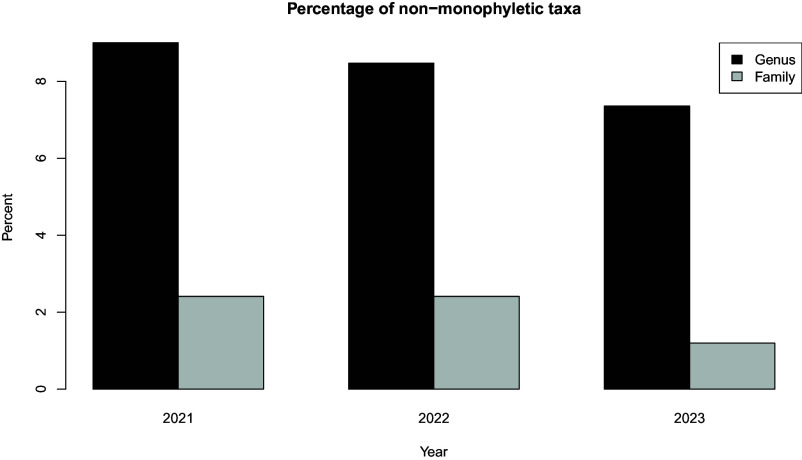
Percentages of taxa, families, and genera, in the Clements taxonomy which are not monophyletic in the phylogenetic synthesis tree, across different taxonomy years.

This synthesis tree largely agrees with the named relationships as captured by the Clements taxonomy. Where there are disagreements, over time, taxonomy is updated to reflect the evolutionary relationships (Fig. 3). For example, this complete tree demonstrates that according to phylogenetic relationships six families, as they were defined in Clements 2021, did not form monophyletic groups; Macrosphenidae, Sarothruridae, Rallidae, Muscicapidae, Turdidae, and Laniidae. However, by the Clements 2023 taxonomy, the membership of Macrosphenidae, Sarothruridae, and Rallidae were corrected to match the current phylogenetic inferences. In the 2024 version of the taxonomy, Muscicapidae and Turdidae were updated to reflect phylogenetic relationships. Laniidae, per the taxonomy, is still not monophyletic because the monotypic family Platylophidae, containing only the Crested Jayshrike (*Platylophus galericulatus*), is nested within it, according to McCullough et al. (24). Other minor conflicts between taxonomy and phylogeny remain. For example around 8% of genera in the Clements taxonomy are not monophyletic according to the phylogenetic synthesis tree (Fig. 3).

### Dates.

We used published date estimates from 120 trees from 90 published studies (full citations are listed in *SI Appendix* Citations, and posted in the data repository). These studies provided date estimates for 7,000 (∼63%) of the internal nodes in the tree. 1,666 nodes had date information provided by only one input study, 1,250 nodes had two input date estimates, and 3,954 had between 3 and 10 input date estimates. There were up to 40 date estimates for other nodes. We had 19 date estimates for the root of the tree, Aves, ranging from 78 Mya (21) to 133 Mya (25). The mean age of the root, Aves, is 99 Mya, which aligns with recent work investigating the divergence time of crown birds (26). The nodes for which there is no published date information are assigned dates equally spaced between the nearest dated parent and child nodes.

While there is high variance among dates across studies (27) [e.g. age estimates for the root vary by up to 55 Mya (21, 25)], and individual dates are approximate, these combined analyses provide a broad view of the timing of divergences across the entire bird tree of life. We generated 100 complete dated trees with topologies stochastically sampled via the taxon addition process, and dated each of these trees using a random sample from the input node age sets as calibration points. For nodes where there was only a single date input, we stochastically sampled or excluded that date with equal probability. We also dated each of these trees using the mean of the input node age sets as calibrations. For each of these samples of 100 dated trees, we summarized these trees into a single majority rule consensus tree topology and branch lengths, with CIs on dates, using RevBayes (28). However, these intervals will underestimate date uncertainty, particularly in regions of the tree with few input date estimates. While the dated tree provides a convenient summary of existing date information across all birds, nearly 40% of nodes have no input date information and are simple interpolations, and therefore individual node dates in the summary tree should be used with caution. Importantly, the individual date estimates for each node, and the metadata linking those estimates back to published studies, is published with the data files for the synthetic tree, so users interested in investigating uncertainty across dates can perform their own downstream analyses. This file can also be used to determine which nodes have input date information, and the inverse, which node dates are solely based on interpolation.

### Custom tree synthesis.

The input phylogenies used here are all publicly available via the Open Tree data store (6). The custom synthesis software is also available both via an API and through a simple browser-based interface (https://aves.opentreeoflife.org/v3/tree_of_life/launch_custom). Any interested user can create their own custom synthesis tree for any set of phylogenies. This synthesis can be performed on trees already present in the Open Tree data store, or by adding new trees to the data store. Phylogenies can be ranked in any preferred order, and a complete synthesis generated by a user for any set of taxa included in the Open Tree taxonomy. We will update the data repository and share new versions of the Aves synthetic tree, at least annually and potentially more often.

### Data attribution.

A key premise of our project is the dynamic but traceable nature of the output. Empowering other researchers to contribute and, critically, be recognized for their own work, is a central goal of the project. Because the provenance of all relationships in the phylogeny are carefully tracked, we are able to readily quantify the proportion of nodes in the final tree that are informed by a given study (*SI Appendix*, Table S1). This remains true for subtrees pruned to represent smaller sets of taxa such as those in a regional study, and we provide easy-to-use code for how to derive the input trees that contribute to the relationships for trees of arbitrary sets of taxa from the larger tree. While we firmly believe that users of our phylogeny should cite all the data sources that inform their work, we recognize that most journals have citation limits, and the proportion of nodes supported by a constituent study can help users to identify the most critical studies to cite. We hope that, in general, this project adds to a growing understanding of the need to adequately track the impact of datasets themselves, which may require changes to journal policy. An important corollary is that, although most journals require data deposition (in large part to facilitate reproducibility), many authors still do not publish the actual tree files they generate and use for downstream analyses (1, 13), and many phylogenies are available only as images in PDFs. This lack of accessible data precludes others from reproducing results and is likewise something we hope that journals address. The data access and attribution pieces go hand in hand, and by building the tools to track these linkages, we aspire to move the needle on these important aspects of the modern scientific process. The Open Tree project not only provides a stable and usable data store, it incentivizes data curation and sharing by offering tools such as custom synthesis and visualizations for consensus and conflict between trees, and between trees and taxonomy (6).

### Interoperability.

**Fig. 4.**
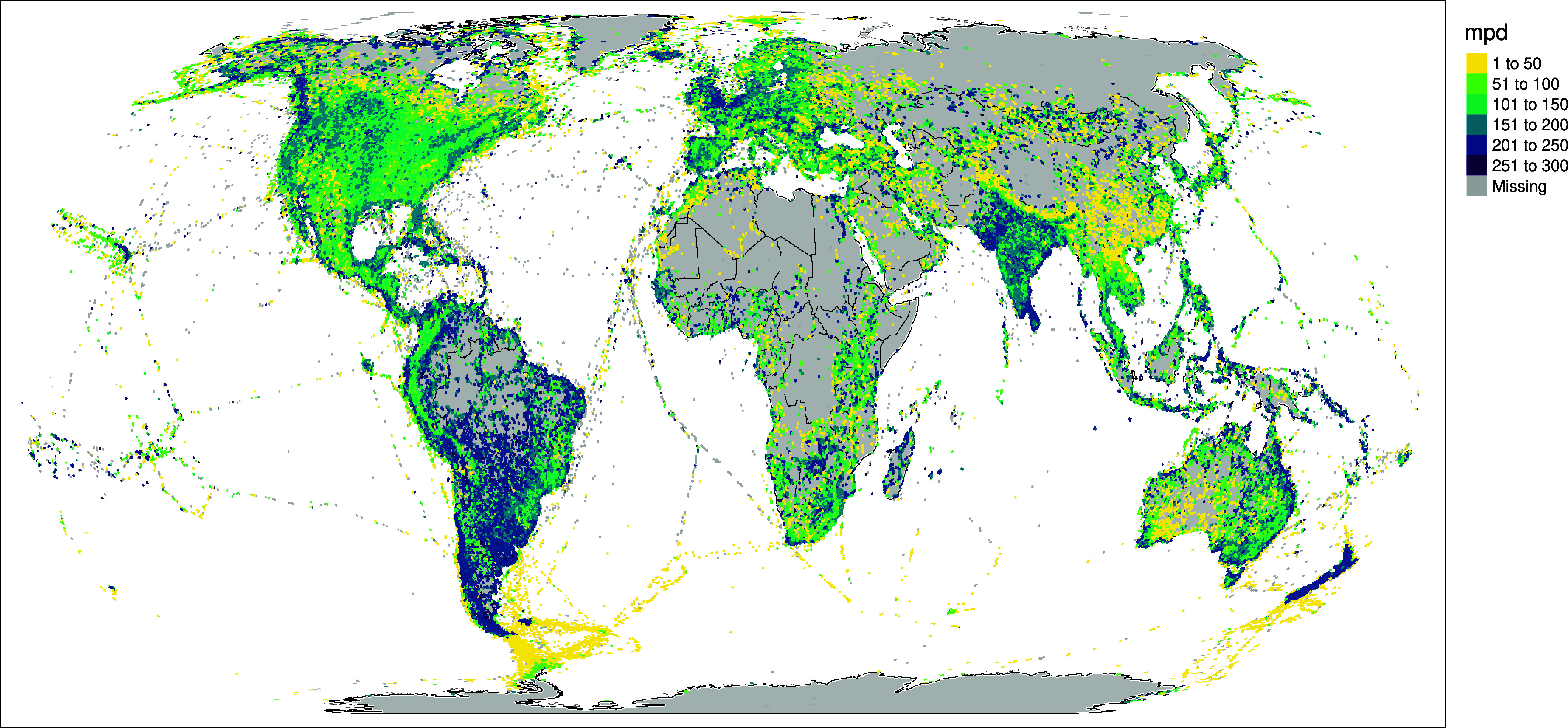
Global phylogenetic diversity of bird records across the globe. Co-occurring bird species tend to be more distantly related in the tropics and in what were once elements of the ancient Gondwanan continent, including Madagascar, New Zealand, South America, and India. Grid cells on the map are colored according to global mean pairwise phylogenetic distance (mpd) among species reported to eBird.

To demonstrate the power of data linkages between this project and others, such as eBird (29), the largest community science project in the world, we downloaded all eBird records, filtered to those we could confidently assign to a precise location, and grouped these into hexagonal grid cells at the global scale. We then calculated the mean pairwise phylogenetic distance among the species in each grid cell, which had the effect of graphically and quantitatively highlighting a number of known but poorly understood global gradients in evolutionary relationships among co-occurring species (Fig. 4). For example, the Andes, particularly at high elevations, are characterized by a phylogenetically clustered set of species when compared to lowland Amazonia. The pattern is even starker in the Himalayas, where over a relatively short geographic distance, the pattern goes from distant relatives co-occurring with one another, to increasingly closely related species co-occurring at higher elevations and into the Tibetan rain shadow (30). Inputs to eBird data vary and are higher in densely populated areas. Data are almost totally absent from some areas and are very dense in others. For example, the linear tracks across the oceans reflect cruise ship routes, and the apparent deep divergences in the United Kingdom likely reflect concerted bird-watching effort there uncovering a bevy of odd vagrant species. Nonetheless the deep evolutionary divergences between species in Madagascar, lower elevations in Papua New Guinea, Southern South American, and Southeast Asia are also visible, and these divergences contrast with the profound phylogenetic clustering of arid and high latitude communities in places such as western Australia (31), the Sahara, and the Southern Ocean. Linking patterns such as these with trait data such as those from AVONET (7) will provide insight into the processes structuring global avian diversity, and such approaches will be greatly facilitated by the interoperability of our project with other data resources like those mentioned here.

## Discussion.

Intrinsically, this synthesis tree approach builds on prior work. We extend and expand the synthesis trees built using earlier versions of the Open Tree synthesis software (32). The efficiency of this software has been improved, allowing for rapid synthesis of even very large trees (33).

Taxonomy is a critical axis for interoperability between projects. In the past, discordance between the taxon names and concepts captured in the Open Tree and the Clements taxonomies has limited ornithological community engagement with Open Tree resources. The automated algorithms required to create a tree of life at the scale of all the world’s organisms result in an avian phylogeny with more taxonomic inconsistencies and less resolution than ornithologists expected. Unifying taxonomic concepts across different resources and through time is nontrivial, and often requires in-depth knowledge of the literature and the history of research on a given taxon. While some automatic name matching is possible, automated systems can also easily lead to errors. Here, via a curated process, we strike a balance between linking data both to large resources such as National Center for Biotechnology Information (NCBI) and Global Biodiversity Information Facility (GBIF), and to taxon concepts in the most up-to-date and accurate bird taxonomic resources (16). This project fills a key gap in current biodiversity informatics—cross-linking names, and unique identifiers associated with those names, at scale. Mapping taxonomic concepts and names across resources in a public, accessible, and reusable database is essential for leveraging the open data resources being generated by the biological community (34).

Each of the tips in our tree are mapped to identifiers and labels in the Open Tree taxonomy and to three different versions of Clements taxonomy, as well as several other taxonomies. This process was facilitated by the use of Avibase taxon concepts (16), and in principle (but not yet in practice) it would be possible to initially curate trees using these taxon concepts, which would further facilitate taxonomic updates and minimize attrition of matched taxa over time.

Our approach also identifies pathways that can be built to link the phylogenetic estimates to the vouchered tissues, physical specimens, and sequence archive records from which they are derived. This interoperability will improve the bioinformatic connections to collections and community science data.

Many birdwatchers keep careful track of which birds they have and have not seen. eBird makes this process fun and easy, and over 800,000 participants have taken advantage of the tool, contributing valuable community science data in the process. This tree directly maps to the species lists used in eBird, and provides an evolutionary framework for analyzing those data (Fig. 4). These links between resources create an opportunity to feed evolutionary information back to birders, capturing the phylogenetic diversity of their observations.

The synthesis approach also affords the opportunity to explore concordance and conflict across estimates of bird relationships through time. While most relationships have remained stable, others are highly contentious (17). By highlighting unstable relationships and species which are absent from our phylogenetic inputs, our framework can help direct future research effort more efficiently.

In our consensus tree, we use a very simple ranking system—relationships in more recent trees are preferred over those in older trees (with the exception of two very large supermatrix trees with relatively high levels of missing data, Burleigh et al. (35) and Jetz et al. (22) which were ranked last). This ranking approach aligns with the notion that, in general, researchers are converging on accurate estimates of phylogenetic relationships. For example, as demonstrated in our exploration of alternate rankings, even when Jetz et al. (22) is moved to the top-ranked spot, only around 14% of branches change. Nonetheless, for some evolutionary questions, alternate topologies might result in different conclusions. To test for sensitivity of conclusions to rankings, users can rerun their analyses on trees synthesized based on alternate rankings or tree sets. This process is straightforward, and all necessary code is available and documented in the AvesTreeCode GitHub repository.

Our supertree and dating methods summarize the information from input studies. Combined with the taxonomic placement step, our pipeline provides a tree with complete sampling of known taxa, and demonstrates how different studies agree or disagree. The results are not intended to be, and indeed cannot be, more accurate than the input trees. Neither most individual phylogenies nor their date estimates are independent estimates (9). Across multiple studies the same sequence data and potentially the same alignments may be reused. Many dated studies may rely on the same fossil calibrations, or on secondary date calibrations from other studies which are also in the set of input studies. In addition, while we sample from node ages estimated across studies, we do not capture the uncertainty intervals for those original estimates. Nonetheless, this is a useful approach for accessing all available published phylogenetic information in a single place.

Our phylogeny will be of use for a variety of research questions, from regional to macroecological studies. Yet, if a high-quality dated tree is available (or can be readily constructed) that encompasses the relevant taxa for a given research question, we would recommend using that tree.

While few groups are as well studied and data-rich as birds, the approaches described here can be applied to unifying taxonomic and phylogenetic information for any group. Although supermatrix-based phylogenies covering extensive diversity across large groups are regularly published, they rarely have complete taxonomic sampling. For example, Zuntini et al. (36) recently published an impressive phylogenomic tree for 8,000 plant taxa using 353 genes. This is an incredible resource which covers 60% of all plant taxa. However, that still leaves 40% of plants unsampled in this tree. Our approach takes advantage of these types of large-scale evolutionary inferences while filling in the gaps using the best phylogenetic information available for the remaining taxa. This synthesis provides an evolutionary framework to harness trait data, phylogenetic inferences, collections data, observation records, and taxonomic concepts, in shareable, reusable, interoperable datastores.

## Materials and Methods

### Methods.

#### Data integration.

We selected and curated 281 trees from 262 published studies containing bird phylogenies published from 1990 through 2024. All of these trees and their associated metadata are now available in the Open Tree data store, phylesystem (6). The full set of citations are posted with the synthesis data at https://github.com/McTavishLab/AvesData and are in *SI Appendix*. All code used for data processing in the study is available and fully documented at https://github.com/McTavishLab/AvesTreeCode. An R package, clootl, for easily accessing the trees and taxonomy files is available at https://github.com/eliotmiller/clootl.

We gathered phylogenetic trees associated with publications from online data stores, and by contacting authors directly. The metadata for each tree was curated to contain standard phylogenetic metadata comprising Minimum information about a Phylogenetic Analysis data (37). These data and metadata are stored in an open data store, in a JavaScript Object Notation format translation of NeXML (38). The Open Tree data store (6) builds upon and incorporates prior work in making phylogenetic data available. Data previously published to Treebase (39) can be directly imported with all metadata. The data store is mirrored to GitHub and available open access. The data store is editable by curators, and all edits and updates are tracked as git commits.

We mapped tips in trees to identifiers in the OTT. The Clements taxonomy is used by the Cornell Lab of Ornithology, which manages eBird, among other data resources. To link taxon names across Clements and OTT, we created a taxonomic translation table which maps every species level taxon in the 2021 Clements taxonomy to a unique identifier in OTT. Via OTT, our translation table links identifiers from the Clements taxonomy to taxon identifiers in NCBI, GBIF, Catalog of Life, and other online taxonomic resources and is used by the Open Tree project (14). In addition, we used the Avibase system to map from the species-level taxonomic concepts in the Clements taxonomy to the taxonomic concepts in the International Ornithological Community World Bird List v14.1 (40), Handbook of the Birds of the World and Birdlife v. 8.1 (41, 43), and the Howard and Moore taxonomy (fourth edition, with corrigenda from 2015) (42). Taxa can be added to OTT via an amendment system. The taxonomic crosswalk is shared with the data resources for this project on GitHub. To match taxon names we used the Open Tree bulk taxon name resolution system, which searches on canonical names and on synonyms stored in OTT https://tree.opentreeoflife.org/curator/tnrs/. Synonyms in OTT are imported from the component taxonomic resources, primarily NCBI and GBIF. Additional matches were made by hand by the authors of this manuscript. Whether matches were made based on “canonical name,” “synonym,” or “hand match” is tracked on the crosswalk table. We added species that were in the Clements taxonomy, but not present in the OTT to OTT via the taxon amendment process. These taxa are available in OTT v 3.5. For each of the input trees, we mapped tips to unique OTT identifiers.

We added some taxonomic constraint trees to reinforce monophyly of higher-level taxa from Clements where there were no direct phylogenetic conflicts with the monophyly of those groups. The Open Tree synthesis algorithm preserves monophyly of taxa where there is no phylogeny directly contesting that relationship, but due to some differences in the membership of groups in the higher taxonomy of the Open Tree taxonomy as compared to the Clements taxonomy, we added constraints capturing those taxonomic groups to ensure those taxa were present in the synthesis tree. All trees used in this analysis are publicly stored in phylesystem in the collection “snacktavish/Aves.”

#### Phylogenetic synthesis.

We ranked the input trees by publication year, with more recent trees ranked higher, with the exception of two large supertrees built from sparse matrices, Burleigh et al. (35) and Jetz et al. (22). We ranked these trees below the other published phylogenies.

We performed custom synthesis using the Open Tree synthesis software. This process works by combining input trees with the taxonomic backbone constructed from a single reference taxonomy using the propinquity and otcetera]Please confirm the spelling of “otcetera.” software developed for the Open Tree of Life project and described in depth in ref. 12. The taxonomy provides a placeholder for taxa without phylogenetic information. Taxon names on input trees are standardized to the same reference taxonomy that is used to construct the taxonomic backbone. This process uses a greedy algorithm that adds branches from input trees in rank order, from the mostly highly ranked tree, to the lowest ranked tree. To make supertree construction tractable, we decompose the overall task into subproblems, based on monophyletic taxa (12). We look for named groups in the pruned taxonomic backbone that are “uncontested” by any single phylogenetic tree. This allows for subdividing the tree in smaller subproblems that will be easier to solve using phylogenetic evidence. To solve the subproblems, there are two steps. First, evidence for each subproblem is identified among exemplified phylogenetic trees and the pruned taxonomic backbone. This is done by pruning away all tips that do not belong to the subproblem from the exemplified phylogenetic trees and the pruned taxonomic backbone. Then, the ranking of phylogenetic trees is used to sequentially gather evidence to resolve relationships in the subproblem. Taxonomic evidence is always ranked last, below any published phylogenetic estimates. Concordance and conflict between each input tree and the resulting estimate is stored, and output as synthetic tree metadata. Recent tooling developments in otcetera (33) have sped up this synthesis process, so that updated synthesis trees for all of Aves (around 11,000 taxa) can be estimated in a few minutes. Every branch in the synthetic tree is annotated with the node in an input study or the taxonomic relationship that supports that branch. These links from input data to each relationship in the synthetic tree make the route from input data to inference clear, and provide a direct path to correcting incorrect inferences.

We created versions of the synthetic tree aligned to the 2021, 2022, and 2023 updates to the Clements taxonomy in our GitHub repository https://github.com/McTavishLab/AvesData.

#### Adding taxa without phylogenetic information.

We prune the synthetic tree to only taxa that are included in input phylogenies, and place the remaining taxa using a curated taxon addition process (Fig. 1 step 4). This process uses a modified version of the addTaxa algorithm (18); https://github.com/eliotmiller/addTaxa], which we describe in brief here. First, missing taxa are identified. “Missing taxa” are any species in the Clements taxonomy for a given year which are not included in the phylogenetic synthesis tree. Second, taxon addition statements are generated for each missing taxon. Each statement takes the form of a family, genus, or species group (i.e. a clade, as identified by the most recent common ancestor of two or more species passed to the algorithm). Multiple addition statements can be provided, and the algorithm will search for the most specific placement possible. For example, in *SI Appendix*, Fig. S1, both members of the genus Poliocephalus are missing from the grebe family tree. The first Poliocephalus is placed at the family-level, while the second is added as sister to the first. Importantly, these taxon addition statements can also contain exclusion statements. Again, using the example in *SI Appendix*, Fig. S1, we specify that Poliocephalus should not break the monophyly of any of the other grebe genera. Third, we implement these taxon statements by randomizing the order missing species are added in, then iterating the process multiple times to generate a cloud of 100 possible, taxonomically complete trees (Fig. 1 step 6). By applying this postprocessing step, we are able to generate from each phylogenetic synthesis tree a distribution of complete, bifurcating, phylogenies, with branch lengths proportional to time.

#### Estimating dates.

There is no branch length information in the custom synthesis tree because the branches are combined estimates from phylogenies generated using a variety of data types and inference types, and some species are added based on taxonomy. We applied date estimates to the complete trees using Chronosynth https://github.com/OpenTreeOfLife/chronosynth. The Chronosynth approach is expanded from the concepts used in Datelife (27), an R package to estimate dated trees using chronograms in the Open Tree data store (6). Chronosynth is written in python and relies on python-opentree as a wrapper for Open Tree API calls (44). Chronosynth summarizes the date information available in the Open Tree datastore, phylesystem (6) by mapping the date estimates from nodes in dated input trees to the nodes in the custom synthetic tree which align with those dated nodes. We map aligning nodes using the tree to tree conflict functionality in otcetera (12). Each internal node in the custom synthetic tree contains a list of source tree nodes that support the synthetic tree node. Imagine pruning the synthetic tree down to have the same terminal set of species as an input tree and suppressing all internal nodes in the induced tree which have only one child node. If one of the internal nodes, X, of this induced tree is the ancestor of the exact same set of species as some internal node, Y, in the source tree, then node X maps to node Y. If the parent of node X is retained in the induced tree (not removed due to having only one child), then we say that X is supported by Y. See the discussion of the “supported_by” annotation in ref. 12 for further details. This alignment step means that when the topology of the custom synthesis tree differs from that of the dated input tree, not all node dates from the dated input can be used in date inference on the complete tree. Dates which are mapped from the input trees to the synthetic tree then are used as secondary node calibrations in the date smoothing algorithm (45). The CIs from input trees are not currently incorporated into Chronosynth estimates. Once nodes are mapped from all of the dated input trees, each node in the custom synth tree may have zero, one, or many dates associated with it. Where multiple dates are available for a node they can be summarized using the mean, or randomly resampled across iterations of summarization.

To sample across some of the uncertainty in dates, we estimated smoothed complete trees for each of our 100 sampled taxon addition trees, using a random sample from the node dates for each node, including the root. The smoothing was performed using bladj in the Phylocom package (45). Bladj fixes the ages of nodes you provide age estimates for and then sets all other branch lengths by placing the nodes evenly between dated nodes, and between dated nodes and terminals.

We share these 100 dated trees in the data repository. We then summarized this set of trees to a single Maximum Clade Credibility tree, limited to positive branch lengths, using RevBayes (28). This places CIs across the nodes. The Maximum Clade Credibility tree is also shared in the data repository. Trees dated using Chronosynth should be considered rough date estimates, which summarize the existing information about node ages across many taxa and studies, rather than providing novel node age information. There are alternate approaches available for smoothing dates that may be more appropriate for some downstream uses, for example by incorporating an explicit birth–death diversification process model when inferring ages for nodes with no date information (18, 46).

#### Taxonomic updates.

We published our data package with the synthesis tree mapped to the Open Tree taxonomy and to the species in Clements taxonomy versions 2021, 2022, and 2023. While most of the taxonomy has remained stable, there have been a few hundred taxon additions and deletions over these three years. We will continue to update and publish new versions of the synthetic tree estimates, as new bird phylogenies are published and added to the data store. In addition, we will share and publish versions of the tree matched to new versions of the Clements taxonomy.

## Supplementary Material

Appendix 01 (PDF)

## Data Availability

Code and input data have been deposited in GitHub (https://github.com/McTavishLab/AvesData and https://github.com/McTavishLab/AvesTreeCode) (47, 48).
