# Supplementary material for: A complete and dynamic tree of birds
Source: Proc Natl Acad Sci U S A. 2025 Apr 29;122(18):e2409658122. doi: 10.1073/pnas.2409658122 (PMC12067227; doi:10.1073/pnas.2409658122)
Supplement: Supplementary file 1 — Appendix 01 (PDF) [file pnas.2409658122.sapp.pdf]

Supplemental Materials:

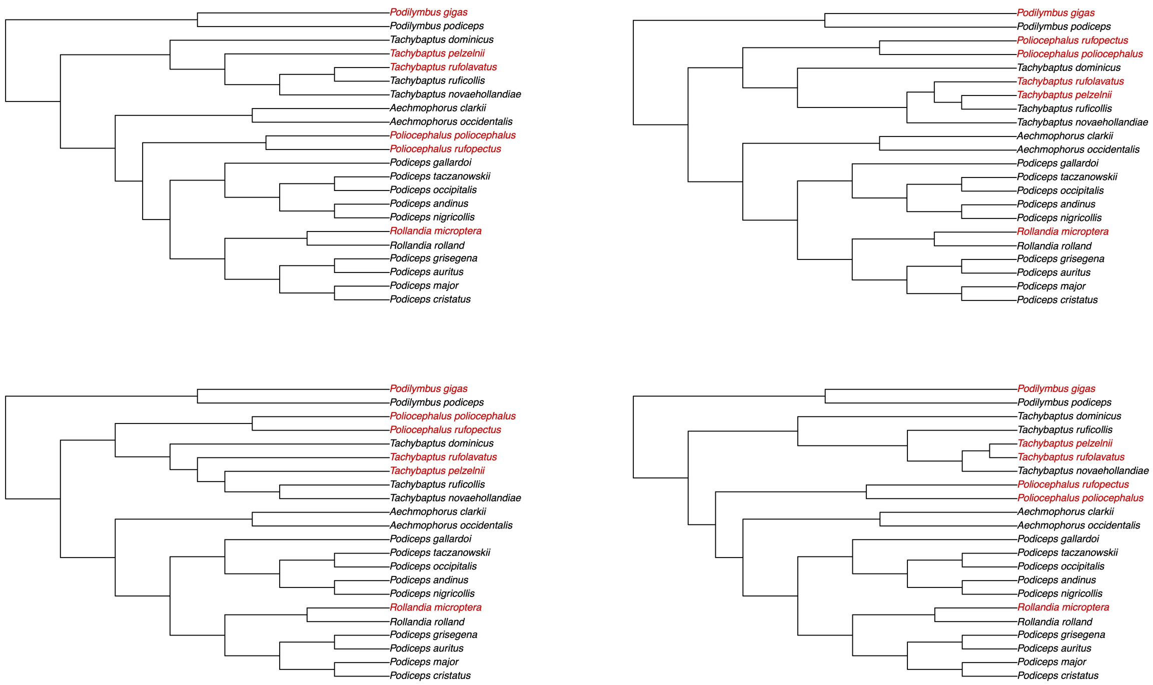

Figure S1: Example trees demonstrating randomization within taxonomic constraints during the taxonomic addition process. Taxonomic constraints were developed from information in Birds of the World

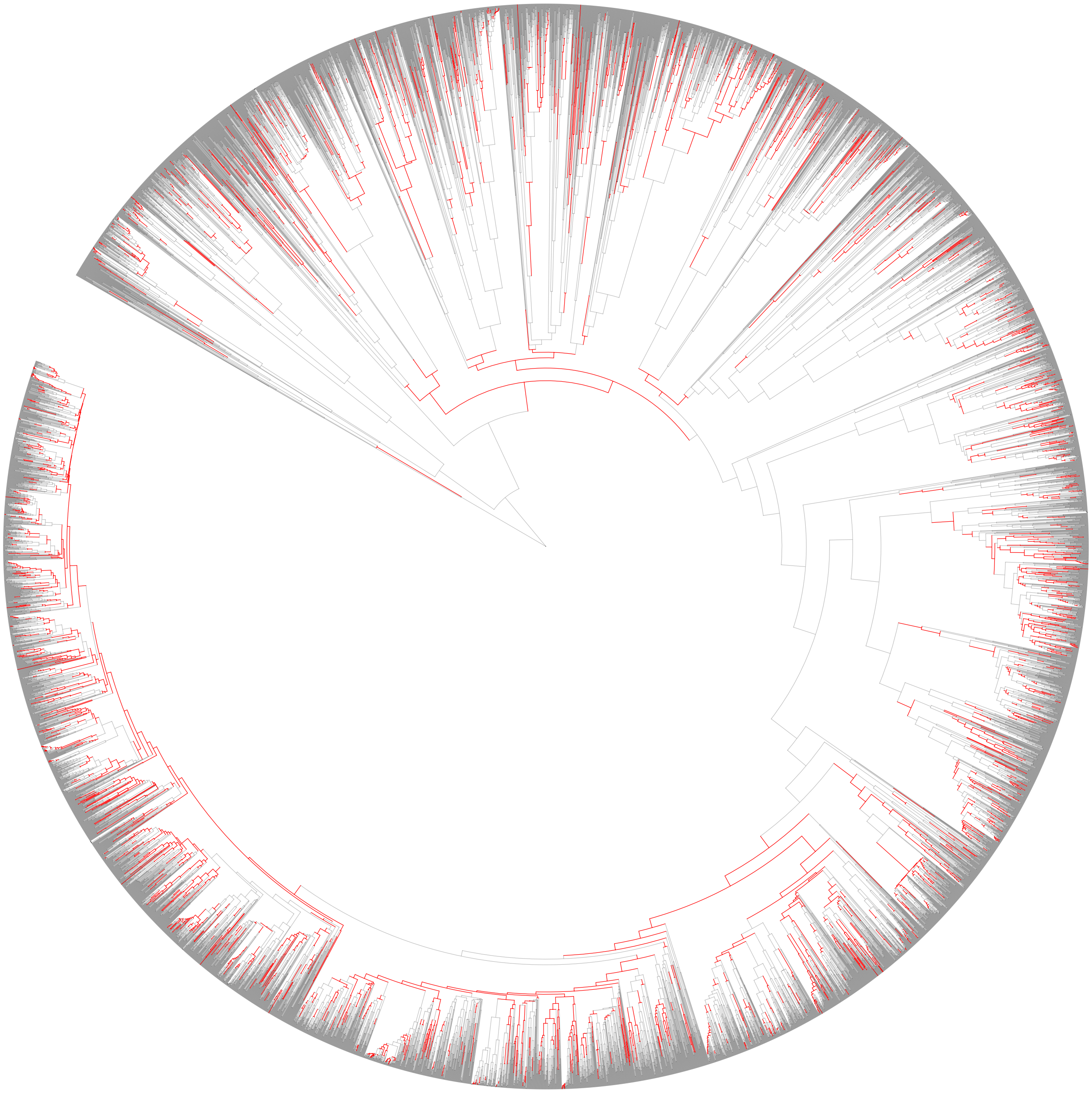

Figure S2: Figure showing Aves 1.3 with branches highlighted in red which conflict with the relationships in Jetz et al. 2012

Table S1. Table showing the proportion of branches in the complete tree supported by each input phylogeny. Column 'used in dating' shows if dates from that study were included in sampling of internal node date estimates.

Proportion of branches	OpenTree studyID	Short Reference	Used in dating
37.02	ot_809	Jetz et al., 2012 [1]	1
36.15	ot_521	Burleigh, Kimball and Braun, 2015 [2]	0
11.21	ot_2015	Harvey et al., 2020 [3]	0
6.63	ot_770	Barker et al., 2015 [4]	1
6.36	ot_2158	McCullough, Moyle, et al., 2019 [5]	1
5.19	ot_290	Selvatti, Gonzaga and Russo, 2015 [6]	1
4.54	ot_520	Jønsson et al., 2016 [7]	1
4.19	ot_2018	Kimball et al., 2019 [8]	1
4.12	ot_2179	Cai et al., 2019 [9]	0
3.31	ot_2145	Smith et al., 2023 [10]	1
3.08	ot_2141	Černý and Natale, 2022 [11]	1
2.92	pg_2829	Burns et al., 2014 [12]	0
2.74	ot_2167	Zhao et al., 2023 [13]	0
2.55	pg_2853	McGuire et al., 2014 [14]	1
2.42	ot_2312	Stiller et al., 2024 [15]	1
2.23	pg_2913	Price et al., 2014 [16]	1

Continued on next page

(Continued)

2.09	ot_1043	Marki et al., 2017 [17]	1
2.08	ot_2143	Päckert et al., 2020 [18]	0
2.04	ot_2079	Kennedy and Spencer, 2014 [19]	1
1.91	ot_2228	Lapiedra et al., 2021 [20]	1
1.89	ot_2017	Shakya and Sheldon, 2017 [21]	0
1.81	pg_1953	Derryberry et al., 2011 [22]	1
1.69	ot_2013	Oliveros, Andersen and Moyle, 2021 [23]	1
1.61	ot_2154	McCullough, Joseph, et al., 2019 [24]	1
1.55	ot_425	Stein, Brown and Mooers, 2015 [25]	1
1.54	ot_2186	Nagy and Tökölyi, 2014 [26]	0
1.47	ot_1055	Reddy set al. 2008 [27]	0
1.38	ot_500	Prum et al., 2015 [28]	1
1.25	ot_531	Claramunt and Cracraft, 2015 [29]	1
1.17	ot_504	Dufort, 2016 [30]	1
1.17	pg_1586	Moyle, 2012 [31]	0
1.16	ot_121	Payne, 2005 [32]	0
1.09	ot_2072	Chen et al., 2021 [33]	0
1.03	pg_2575	Barker et al., 2013 [34]	1
1.03	pg_2804	Kimball, Mary and Braun, 2011 [35]	0

Continued on next page

(Continued)

1	ot_1170	Shakya et al., 2017 [36]	0
0.98	ot_2172	De Silva, Peterson and Perktas, 2019 [37]	0
0.95	ot_2222	Olsson and Alström, 2020 [38]	0
0.87	pg_2591	Lovette et al., 2010 [39]	1
0.86	pg_2924	Klicka et al., 2014 [40]	0
0.84	pg_2707	Powell et al., 2014 [41]	0
0.83	ot_180	Lovette and Rubenstein, 2007 [42]	0
0.79	ot_2230	Alström et al., 2023 [43]	1
0.79	ot_863	Scofield et al., 2017 [44]	1
0.78	ot_116	Hugall and Stuart-Fox, 2012 [45]	0
0.72	pg_2860	Fulton, Letts and Shapiro, 2012 [46]	0
0.71	pg_2692	Zuccon et al., 2012 [47]	0
0.67	pg_2866	Gonzalez et al., 2013 [48]	0
0.67	ot_2231	Jha et al., 2021 [49]	1
0.67	ot_2307	Wu et al., 2024 [50]	0
0.66	ot_783	Moyle et al., 2016 [51]	1
0.64	ot_1002	Hosner, Braun and Kimball, 2016 [52]	0
0.61	ot_1287	Alström et al., 2018 [53]	1
0.6	pg_2404	Barker et al., 2004 [54]	0

Continued on next page

(Continued)

0.58	ot_111	Alström et al., 2014 [55]	0
0.58	ot_753	Wang et al., 2017 [56]	1
0.57	pg_2416	Nunn and Stanley, 1998 [57]	0
0.56	ot_412	Barker, 2014 [58]	0
0.56	ot_2090	Fuchs, Johnson and Mindell, 2015 [59]	1
0.56	ot_2108	Oliver et al., 2020 [60]	1
0.55	ot_816	Gibson and Baker, 2012 [61]	0
0.55	pg_2869	Gonzalez, Düttmann and Wink, 2009 [62]	0
0.54	pg_2599	Alström et al., 2013 [63]	0
0.52	ot_2180	Oliveros et al., 2019 [64]	0
0.51	ot_2178	Alström et al., 2018 [65]	1
0.49	ot_178	Sheldon et al., 2005 [66]	0
0.48	ot_2097	Kirchman et al., 2022 [67]	0
0.47	ot_2170	Hosner et al., 2016 [68]	1
0.44	ot_782	Beresford et al., 2005 [69]	0
0.44	pg_2600	Johansson, Fjeldså and Bowie, 2008 [70]	0
0.43	ot_2314	Ostrow et al., 2023 [71]	0
0.41	ot_2082	Cai et al., 2021 [72]	1
0.41	pg_2872	Han, Robbins and Braun, 2010 [73]	0

Continued on next page

(Continued)

0.4	ot_2159	Batista et al., 2020 [74]	1
0.4	ot_2221	Hruska et al., 2023 [75]	0
0.38	ot_2160	Garcia-R et al., 2020 [76]	0
0.38	ot_2085	Salter et al., 2020 [77]	0
0.37	ot_532	Gibb et al., 2015 [78]	1
0.37	ot_2151	Pietersen et al., 2019 [79]	0
0.37	ot_2234	Shakya et al., 2020 [80]	0
0.36	ot_2156	Andersen et al., 2019 [81]	0
0.36	pg_2876	Bertelli and Porzecanski, 2004 [82]	0
0.36	ot_2270	Davies, 2015 [83]	0
0.34	ot_854	Johansson et al., 2013 [84]	0
0.34	ot_773	Wood et al., 2016 [85]	0
0.32	pg_2858	Nylander et al., 2008 [86]	0
0.32	ot_2169	Olsson et al., 2013 [87]	0
0.32	pg_2805	Wright et al., 2008 [88]	0
0.31	ot_2152	Cicero et al., 2020 [89]	0
0.31	ot_2188	Nagy, Végvári and Varga, 2019 [90]	0
0.31	ot_2125	Stervander et al., 2020 [91]	0
0.3	ot_763	Irestedt et al., 2008 [92]	0
0.3	ot_140	Kennedy et al., 2022 [93]	0
0.3	pg_1854	Mann et al., 2006 [94]	0

Continued on next page

(Continued)

0.29	ot_2183	Salter et al., 2022 [95]	0
0.28	ot_150	Den Tex and Leonard, 2013 [96]	0
0.28	ot_794	Fuchs et al., 2007 [97]	0
0.28	pg_2806	Joseph et al., 2011 [98]	0
0.27	ot_103	Arbabi, Gonzalez and Wink, 2014 [99]	1
0.27	ot_159	Benz and Robbins, 2011 [100]	0
0.27	ot_2146	Gibb and Shepherd, 2022 [101]	1
0.27	ot_2080	Imfeld, Barker and Brumfield, 2020 [102]	1
0.27	pg_1887	Lerner et al., 2011 [103]	0
0.27	ot_179	Lovette et al., 2012 [104]	0
0.26	ot_1997	Bridge, Jones and Baker, 2005 [105]	1
0.26	ot_156	Moyle, 2004 [106]	0
0.26	ot_843	Rheindt, Norman and Christidis, 2008 [107]	0
0.24	pg_2850	Cibois, Thibault and Pasquet, 2008 [108]	1
0.24	ot_749	Hooper, Olsson and Alström, 2016 [109]	0
0.23	ot_837	Moyle, 2006 [110]	0
0.23	pg_2796	Ohlson, Fjeldså and Ericson, 2013 [111]	0

Continued on next page

(Continued)

0.23	ot_147	Ornelas, González and Espinosa De Los Monteros, 2009 [112]	0
0.22	ot_832	Moyle and Marks, 2006 [113]	0
0.22	ot_841	Treplin et al., 2008 [114]	0
0.21	ot_757	Ericson et al., 2020 [115]	0
0.21	ot_312	Schweizer et al., 2015 [116]	1
0.2	ot_112	Päckert, Martens, Sun, et al., 2012 [117]	0
0.19	ot_289	Dos Remedios et al., 2015 [118]	0
0.19	pg_2875	Jønsson et al., 2010 [119]	0
0.19	pg_2015	Ödeen, Håstad and Alström, 2011 [120]	0
0.19	pg_2444	Pereira et al., 2007 [121]	0
0.19	ot_124	Pitra et al., 2002 [122]	0
0.19	ot_874	Zuccon et al., 2006 [123]	0
0.18	ot_2182	Alström et al., 2011 [124]	1
0.18	ot_2191	Alström et al., 2015 [125]	0
0.18	ot_835	Moyle, Cracraft, et al., 2007 [126]	0
0.18	ot_174	Nyári et al., 2009 [127]	0
0.18	ot_2104	Ericson et al., 2014 [128]	1
0.18	pg_2845	Pasquet et al., 2014 [129]	0
0.18	ot_144	Pereira and Baker, 2008 [130]	0
0.18	ot_161	Russello and Amato, 2004 [131]	0

Continued on next page

(Continued)

0.18	ot_768	Slager et al., 2014 [132]	0
0.17	ot_867	Fuchs et al., 2008 [133]	0
0.17	ot_118	Harris, Carling and Lovette, 2014 [134]	0
0.17	pg_1966	Lee, Joseph and Edwards, 2012 [135]	0
0.17	ot_148	Marks, Weckstein and Moyle, 2007 [136]	0
0.17	ot_838	Moyle, 2005 [137]	0
0.17	pg_2874	Tietze et al., 2013 [138]	0
0.16	pg_2454	Brumfield and Edwards, 2007 [139]	0
0.16	ot_533	Dantas et al., 2016 [140]	1
0.16	ot_129	Njabo and Sorenson, 2009 [141]	0
0.15	ot_2084	Bryson et al., 2016 [142]	0
0.15	ot_848	Lovette et al., 2008 [143]	0
0.15	ot_1286	Smith et al., 2018 [144]	1
0.15	ot_2132	White and Braun, 2019 [145]	0
0.14	ot_2091	Buainain et al., 2022 [146]	1
0.14	ot_831	Moyle, Chesser, et al., 2006 [147]	0
0.14	ot_731	Ottenburghs et al., 2016 [148]	1
0.13	ot_655	Besnard et al., 2016 [149]	0
0.13	ot_2173	Campillo et al., 2018 [150]	0

Continued on next page

(Continued)

0.13	pg_2871	Fuchs, Johnson and Mindell, 2012 [151]	0
0.13	ot_806	Gavryushkina et al., 2016 [152]	1
0.13	ot_862	Zhou et al., 2016 [153]	0
0.12	ot_1022	Donne-Goussé, Laudet and Hänni, 2002 [154]	0
0.12	ot_866	Gonzalez and Wink, 2008 [155]	0
0.12	ot_126	Krajewski, Sipiorski and Anderson, 2010 [156]	0
0.12	ot_2187	Nováková and Robovský, 2021 [157]	0
0.12	ot_138	Slikas, 1997 [158]	0
0.12	ot_2020	Vianna et al., 2020 [159]	1
0.11	ot_169	Bryson et al., 2014 [160]	0
0.11	ot_142	Chambers et al., 2009 [161]	0
0.11	ot_177	Dor et al., 2010 [162]	0
0.11	pg_1872	Fain, Krajewski and Houde, 2007 [163]	0
0.11	ot_855	Irestedt et al., 2009 [164]	0
0.11	ot_786	Ksepka et al., 2012 [165]	0
0.11	ot_166	Quintero, Ribas and Cracraft, 2013 [166]	0
0.11	pg_2332	Voelker, 2002 [167]	0
0.09	ot_2168	Moltesen et al., 2012 [168]	0

Continued on next page

(Continued)

0.09	ot_769	Sweet and Johnson, 2015 [169]	1
0.08	ot_101	Chaves, Hidalgo and Klicka, 2013 [170]	0
0.08	pg_2870	Fuchs et al., 2011 [171]	0
0.08	ot_415	García–R, Gibb and Trewick, 2014 [172]	1
0.08	ot_1285	Johansson et al., 2018 [173]	0
0.08	ot_113	Lerner, Klaver and Mindell, 2008 [174]	0
0.08	ot_2155	McCullough et al., 2022 [175]	0
0.08	ot_836	Moyle, et al., 2006 [176]	0
0.08	ot_305	Oatley, Simmons and Fuchs, 2015 [177]	0
0.08	ot_139	Ramirez, Miyaki and Del Lama, 2013 [178]	0
0.08	ot_2185	Younger et al., 2019 [179]	0
0.07	ot_2268	Garcia-R. and Trewick, 2015 [180]	1
0.07	ot_2074	Johnson, Howard and Brumfield, 2021 [181]	1
0.07	ot_2148	Liu et al., 2017 [182]	1
0.07	ot_172	Päckert, Martens, Wink, et al., 2012 [183]	0
0.07	ot_153	Patel et al., 2011 [184]	0

Continued on next page

(Continued)

0.07	ot_137	Patterson, Morris-Pocock and Friesen, 2011 [185]	0
0.07	ot_830	Pereira and Wajntal, 2008 [186]	0
0.07	ot_120	Pereira and Baker, 2004 [187]	0
0.07	ot_160	White et al., 2011 [188]	0
0.07	ot_2083	Yonezawa et al., 2017 [189]	1
0.07	ot_2190	Zhang et al., 2016 [190]	0
0.06	ot_157	Benz, Robbins and Peterson, 2006 [191]	0
0.06	ot_176	Cerasale et al., 2012 [192]	0
0.06	ot_891	Lamichhaney et al., 2015 [193]	1
0.06	ot_154	Lutz et al., 2013 [194]	0
0.06	ot_158	Moore, Overton and Miglia, 2011 [195]	0
0.06	ot_167	Ribas, Miyaki and Cracraft, 2009 [196]	0
0.06	pg_2890	Rice, 2005 [197]	0
0.06	ot_171	Spellman et al., 2008 [198]	0
0.06	ot_880	Zink and Blackwell, 1998 [199]	0
0.06	ot_820	Zino, Brown and Biscoito, 2008 [200]	0
0.06	ot_894	Aliabadian et al., 2007 [201]	0
0.06	ot_914	Cibois et al., 2014 [202]	0
0.06	pg_1764	Kennedy et al., 2013 [203]	0

Continued on next page

(Continued)

0.06	ot_834	Moyle, et al., 2008 [204]	0
0.06	ot_175	Nguembock et al., 2008 [205]	0
0.06	ot_119	Pereira, Baker and Wajntal, 2002 [206]	0
0.06	ot_164	Ribas, Joseph and Miyaki, 2006 [207]	0
0.06	ot_162	Schweizer, Güntert and Hertwig, 2012 [208]	0
0.05	ot_873	Blechschmidt, et al., 1993 [209]	0
0.05	ot_173	Harris, Birks and Leaché, 2014 [210]	0
0.05	ot_812	Johnson et al., 2016 [211]	1
0.05	ot_2098	Moncrieff, Faircloth and Brumfield, 2022 [212]	0
0.05	ot_110	Schweizer and Shirihai, 2013 [213]	1
0.05	ot_165	Smith et al., 2013 [214]	0
0.05	pg_2798	Subramanian et al., 2013 [215]	1
0.04	ot_2192	Alström et al., 2020 [216]	0
0.04	ot_2189	Alström, Rasmussen, et al., 2018 [217]	1
0.04	ot_151	Armenta, Weckstein and Lane, 2005 [218]	0
0.04	ot_155	Bonaccorso et al., 2011 [219]	0

Continued on next page

(Continued)

0.04	ot_2184	Bruxaux et al., 2018 [220]	0
0.04	pg_2864	Chesser et al., 2010 [221]	0
0.04	ot_869	Dumbacher et al., 2008 [222]	0
0.04	ot_123	Dumbacher, 2003 [223]	0
0.04	ot_2153	Milá et al., 2021 [224]	1
0.04	pg_2926	Mitchell et al., 2014 [225]	1
0.04	ot_170	Päckert, Martens and Severinghaus, 2009 [226]	0
0.04	ot_152	Patané et al., 2009 [227]	0
0.04	ot_313	Shipham et al., 2015 [228]	1
0.04	ot_122	Torres et al., 2014 [229]	1
0.04	ot_1662	White, Mitter and Braun, 2017 [230]	0
0.04	ot_136	Whittingham, Sheldon and Emlen, 2000 [231]	0
0.03	ot_2220	Alström, Rasmussen, et al., 2016 [232]	1
0.03	ot_104	Boertmann, 1990 [233]	0
0.03	ot_856	Illera et al., 2008 [234]	0
0.03	ot_847	Manegold, 2008 [235]	0
0.03	ot_846	Melo and Fuchs, 2008 [236]	0
0.03	ot_864	Ogawa et al., 2015 [237]	1
0.03	ot_102	Pulgarín-R et al., 2013 [238]	0

Continued on next page

(Continued)

0.03	ot_2147	Sprengelmeyer, 2014 [239]	0
0.03	ot_2149	Voelker and Light, 2011 [240]	0
0.03	ot_815	Zwiers, Borgia and Fleischer, 2008 [241]	0
0.02	pg_2857	Arshad et al., 2009 [242]	0
0.02	ot_2181	Johnson et al., 2021 [243]	0
0.02	ot_2150	Kennedy et al., 2019 [244]	0
0.02	pg_2105	Lecroy and Barker, 2006 [245]	0
0.02	ot_747	Mitchell et al., 2016 [246]	1
0.02	ot_824	Reddy et al., 2017 [247]	0
0.02	pg_1979	Tavares et al., 2006 [248]	0
0.02	ot_882	Zink et al., 1999 [249]	0
0.01	ot_2193	Alström, Rasmussen, et al., 2021 [250]	0
0.01	pg_2371	Corl and Ellegren, 2013 [251]	1
0.01	ot_870	Feinstein, Yang and Li, 2008 [252]	0
0.01	ot_125	Ribas et al., 2012 [253]	1
0.01	ot_822	Woog et al., 2008 [254]	0
0	pg_2702	Aggerbeck et al., 2014 [255]	1
0	ot_2122	Alström et al., 2021 [256]	0
0	ot_146	Baker, Pereira and Paton, 2007 [257]	0
0	pg_2865	García-Deras et al., 2008 [258]	0

Continued on next page

(Continued)

0	ot_853	Jønsson et al., 2008 [259]	0
0	ot_149	Overton and Rhoads, 2004 [260]	0
0	ot_827	Parchman, Benkman and Mezquida, 2007 [261]	0
0	ot_823	Scholes III, 2008 [262]	0

References

[1] W Jetz, GH Thomas, JB Joy, K Hartmann, AO Mooers, The global diversity of birds in space and time. *Nature* **491**, 444–448 (2012).

[2] JG Burleigh, RT Kimball, EL Braun, Building the avian tree of life using a large-scale, sparse supermatrix. *Molecular Phylogenetics and Evolution* **84**, 53–63 (2015).

[3] MG Harvey, et al., The evolution of a tropical biodiversity hotspot. *Science* **370**, 1343–1348 (2020).

[4] FK Barker, KJ Burns, J Klicka, SM Lanyon, IJ Lovette, New insights into New World biogeography: An integrated view from the phylogeny of blackbirds, cardinals, sparrows, tanagers, warblers, and allies. *The Auk* **132**, 333–348 (2015).

[5] JM McCullough, RG Moyle, BT Smith, MJ Andersen, A Laurasian origin for a pantropical bird radiation is supported by genomic and fossil data (Aves: Coraciiformes). *Proceedings of the Royal Society B: Biological Sciences* **286**, 20190122 (2019).

[6] AP Selvatti, LP Gonzaga, CADM Russo, A Paleogene origin for crown passerines and the diversification of the Oscines in the New World. *Molecular Phylogenetics and Evolution* **88**, 1–15 (2015).

[7] KA Jønsson, et al., A supermatrix phylogeny of corvoid passerine birds (Aves: Corvides). *Molecular Phylogenetics and Evolution* **94**, 87–94 (2016).

[8] RT Kimball, et al., A Phylogenomic Supertree of Birds. *Diversity* **11**, 109 (2019).

[9] T Cai, et al., Near-complete phylogeny and taxonomic revision of the world's babblers (Aves: Passeriformes). *Molecular Phylogenetics and Evolution* **130**, 346–356 (2019).

[10] BT Smith, et al., Phylogenomic Analysis of the Parrots of the World Distinguishes Artifactual from Biological Sources of Gene Tree Discordance. *Systematic Biology* **72**, 228–241 (2023).

[11] D Černý, R Natale, Comprehensive taxon sampling and vetted fossils help clarify the time tree of shorebirds (Aves, Charadriiformes). *Molecular Phylogenetics and Evolution* **177**, 107620 (2022).

[12] KJ Burns, et al., Phylogenetics and diversification of tanagers (Passeriformes: Thraupidae), the largest radiation of Neotropical songbirds. *Molecular Phylogenetics and Evolution* **75**, 41–77 (2014).

[13] M Zhao, J Gordon Burleigh, U Olsson, P Alström, RT Kimball, A near-complete and time-calibrated phylogeny of the Old World flycatchers, robins and chats (Aves, Muscicapidae). *Molecular Phylogenetics and Evolution* **178**, 107646 (2023).

[14] J McGuire, et al., Molecular Phylogenetics and the Diversification of Hummingbirds. *Current Biology* **24**, 910–916 (2014).

[15] J Stiller, et al., Complexity of avian evolution revealed by family-level genomes. *Nature* (2024).

[16] TD Price, et al., Niche filling slows the diversification of Himalayan songbirds. *Nature* **509**, 222–225 (2014).

[17] PZ Marki, et al., Supermatrix phylogeny and biogeography of the Australasian Meliphagides radiation (Aves: Passeriformes). *Molecular Phylogenetics and Evolution* **107**, 516–529 (2017).

[18] M Päckert, et al., "Into and Out of" the Qinghai-Tibet Plateau and the Himalayas: Centers of origin and diversification across five clades of Eurasian montane and alpine passerine birds. *Ecology and Evolution* **10**, 9283–9300 (2020).

[19] M Kennedy, HG Spencer, Classification of the cormorants of the world. *Molecular Phylogenetics and Evolution* **79**, 249–257 (2014).

[20] O Lapiedra, F Sayol, J Garcia-Porta, D Sol, Niche shifts after island colonization spurred adaptive diversification and speciation in a cosmopolitan bird clade. *Proceedings of the Royal Society B: Biological Sciences* **288**, 20211022 (2021).

[21] SB Shakya, J Fuchs, JM Pons, FH Sheldon, Tapping the woodpecker tree for evolutionary insight. *Molecular Phylogenetics and Evolution* **116**, 182–191 (2017).

[22] EP Derryberry, et al., Lineage Diversification and Morphological Evolution in a Large-Scale Continental Radiation: The Neotropical Ovenbirds and Woodcreepers (aves: Furnariidae): Diversification of a Continental Radiation. *Evolution* **65**, 2973–2986 (2011).

[23] CH Oliveros, MJ Andersen, RG Moyle, A phylogeny of white-eyes based on ultraconserved elements. *Molecular Phylogenetics and Evolution* **164**, 107273 (2021).

[24] JM McCullough, L Joseph, RG Moyle, MJ Andersen, Ultraconserved elements put the final nail in the coffin of traditional use of the genus *Meliphaga* (Aves: Meliphagidae). *Zoologica Scripta* **48**, 411–418 (2019).

[25] RW Stein, JW Brown, Aooers, A molecular genetic time scale demonstrates Cretaceous origins and multiple diversification rate shifts within the order Galliformes (Aves). *Molecular Phylogenetics and Evolution* **92**, 155–164 (2015).

[26] J Nagy, J Tökölyi, Phylogeny, Historical Biogeography and the Evolution of Migration in Accipitrid Birds of Prey (Aves: Accipitriformes). *Ornis Hungarica* **22**, 15–35 (2014).

[27] S Reddy, Systematics and biogeography of the shrike-babblers (Pteruthius): Species limits, molecular phylogenetics, and diversification patterns across southern Asia. *Molecular Phylogenetics and Evolution* **47**, 54–72 (2008).

[28] RO Prum, et al., A comprehensive phylogeny of birds (Aves) using targeted next-generation DNA sequencing. *Nature* **526**, 569–573 (2015).

[29] S Claramunt, J Cracraft, A new time tree reveals Earth history's imprint on the evolution of modern birds. *Science Advances* **1**, e1501005 (2015).

[30] MJ Dufort, An augmented supermatrix phylogeny of the avian family Picidae reveals uncertainty deep in the family tree. *Molecular Phylogenetics and Evolution* **94**, 313–326 (2016).

[31] RG Moyle, MJ Andersen, CH Oliveros, FD Steinheimer, S Reddy, Phylogeny and Biogeography of the Core Babblers (Aves: Timaliidae). *Systematic Biology* **61**, 631–651 (2012).

[32] RB Payne, *The Cuckoos*, Bird Families of the World. (Oxford University Press, Oxford, New York), (2005).

[33] D Chen, et al., Divergence time estimation of Galliformes based on the best gene shopping scheme of ultraconserved elements. *BMC Ecology and Evolution* **21**, 209 (2021).

[34] FK Barker, KJ Burns, J Klicka, SM Lanyon, IJ Lovette, Going to Extremes: Contrasting Rates of Diversification in a Recent Radiation of New World Passerine Birds. *Systematic Biology* **62**, 298–320 (2013).

[35] RT Kimball, CMS Mary, EL Braun, A Macroevolutionary Perspective on Multiple Sexual Traits in the Phasianidae (Galliformes). *International Journal of Evolutionary Biology* **2011**, 1–16 (2011).

[36] SB Shakya, FH Sheldon, The phylogeny of the world's bulbuls (Pycnonotidae) inferred using a supermatrix approach. *Ibis* **159**, 498–509 (2017).

[37] TN De Silva, AT Peterson, U Perktas, An extensive molecular phylogeny of weaverbirds (Aves: Ploceidae) unveils broad nonmonophyly of traditional genera and new relationships. *The Auk* **136**, ukz041 (2019).

[38] U Olsson, P Alström, A comprehensive phylogeny and taxonomic evaluation of the waxbills (Aves: Estrildidae). *Molecular Phylogenetics and Evolution* **146**, 106757 (2020).

[39] IJ Lovette, et al., A comprehensive multilocus phylogeny for the wood-warblers and a revised classification of the Parulidae (Aves). *Molecular Phylogenetics and Evolution* **57**, 753–770 (2010).

[40] J Klicka, et al., A comprehensive multilocus assessment of sparrow (Aves: Passerellidae) relationships. *Molecular Phylogenetics and Evolution* **77**, 177–182 (2014).

[41] AF Powell, et al., A comprehensive species-level molecular phylogeny of the New World blackbirds (Icteridae). *Molecular Phylogenetics and Evolution* **71**, 94–112 (2014).

[42] IJ Lovette, DR Rubenstein, A comprehensive molecular phylogeny of the starlings (Aves: Sturnidae) and mockingbirds (Aves: Mimidae): Congruent mtDNA and nuclear trees for a cosmopolitan avian radiation. *Molecular Phylogenetics and Evolution* **44**, 1031–1056 (2007).

[43] P Alström, et al., Systematics of the avian family Alaudidae using multilocus and genomic data. *Avian Research* **14**, 100095 (2023).

[44] RP Scofield, et al., The origin and phylogenetic relationships of the New Zealand ravens. *Molecular Phylogenetics and Evolution* **106**, 136–143 (2017).

[45] AF Hugall, D Stuart-Fox, Accelerated speciation in colour-polymorphic birds. *Nature* **485**, 631–634 (2012).

[46] TL Fulton, B Letts, B Shapiro, Multiple losses of flight and recent speciation in steamer ducks. *Proceedings of the Royal Society B: Biological Sciences* **279**, 2339–2346 (2012).

[47] D Zuccon, R Prŷs-Jones, PC Rasmussen, PG Ericson, The phylogenetic relationships and generic limits of finches (Fringillidae). *Molecular Phylogenetics and Evolution* **62**, 581–596 (2012).

[48] JCT Gonzalez, BC Sheldon, NJ Collar, JA Tobias, A comprehensive molecular phylogeny for the hornbills (Aves: Bucerotidae). *Molecular Phylogenetics and Evolution* **67**, 468–483 (2013).

[49] A Jha, S Seneviratne, HS Prayag, K Vasudevan, Phylogeny identifies multiple colonisation events and Miocene aridification as drivers of South Asian bulbul (Passeriformes: Pycnonotidae) diversification. *Organisms Diversity & Evolution* **21**, 783–794 (2021).

[50] S Wu, et al., Genomes, fossils, and the concurrent rise of modern birds and flowering plants in the Late Cretaceous. *Proceedings of the National Academy of Sciences* **121**, e2319696121 (2024).

[51] RG Moyle, et al., Tectonic collision and uplift of Wallacea triggered the global songbird radiation. *Nature Communications* **7**, 12709 (2016).

[52] PA Hosner, BC Faircloth, TC Glenn, EL Braun, RT Kimball, Avoiding Missing Data Biases in Phylogenomic Inference: An Empirical Study in the Landfowl (Aves: Galliformes). *Molecular Biology and Evolution* **33**, 1110–1125 (2016).

[53] P Alström, et al., Complete species-level phylogeny of the leaf warbler (Aves: Phylloscopidae) radiation. *Molecular Phylogenetics and Evolution* **126**, 141–152 (2018).

[54] FK Barker, A Cibois, P Schikler, J Feinstein, J Cracraft, Phylogeny and diversification of the largest avian radiation. *Proceedings of the National Academy of Sciences* **101**, 11040–11045 (2004).

[55] P Alström, et al., Discovery of a relict lineage and monotypic family of passerine birds. *Biology Letters* **10**, 20131067 (2014).

[56] N Wang, RT Kimball, EL Braun, B Liang, Z Zhang, Ancestral range reconstruction of Galliformes: the effects of topology and taxon sampling. *Journal of Biogeography* **44**, 122–135 (2017).

[57] GB Nunn, SE Stanley, Body size effects and rates of cytochrome b evolution in tube-nosed seabirds. *Molecular Biology and Evolution* **15**, 1360–1371 (1998).

[58] FK Barker, Mitogenomic data resolve basal relationships among passeriform and passeridan birds. *Molecular Phylogenetics and Evolution* **79**, 313–324 (2014).

[59] J Fuchs, JA Johnson, DP Mindell, Rapid diversification of falcons (Aves: Falconidae) due to expansion of open habitats in the Late Miocene. *Molecular Phylogenetics and Evolution* **82**, 166–182 (2015).

[60] PM Oliver, H Heiniger, AF Hugall, L Joseph, KJ Mitchell, Oligocene divergence of frogmouth birds (Podargidae) across Wallace's Line. *Biology Letters* **16**, 20200040 (2020).

[61] R Gibson, A Baker, Multiple gene sequences resolve phylogenetic relationships in the shorebird suborder Scolopaci (Aves: Charadriiformes). *Molecular Phylogenetics and Evolution* **64**, 66–72 (2012).

[62] J Gonzalez, H Düttmann, M Wink, Phylogenetic relationships based on two mitochondrial genes and hybridization patterns in Anatidae. *Journal of Zoology* **279**, 310–318 (2009).

[63] P Alström, et al., Multilocus phylogeny of the avian family Alaudidae (larks) reveals complex morphological evolution, non-monophyletic genera and hidden species diversity. *Molecular Phylogenetics and Evolution* **69**, 1043–1056 (2013).

[64] CH Oliveros, et al., Earth history and the passerine superradiation. *Proceedings of the National Academy of Sciences* **116**, 7916–7925 (2019).

[65] P Alström, et al., Comprehensive molecular phylogeny of the grassbirds and allies (Locustellidae) reveals extensive non-monophyly of traditional genera, and a proposal for a new classification. *Molecular Phylogenetics and Evolution* **127**, 367–375 (2018).

[66] FH Sheldon, LA Whittingham, RG Moyle, B Slikas, DW Winkler, Phylogeny of swallows (Aves: Hirundinidae) estimated from nuclear and mitochondrial DNA sequences. *Molecular Phylogenetics and Evolution* **35**, 254–270 (2005).

[67] JJ Kirchman, et al., Corrigendum to: Phylogeny based on ultraconserved elements clarifies the evolution of rails and allies (Ralloidea) and is the basis for a revised classification. *Ornithology* **139**, ukab065 (2022).

[68] PA Hosner, EL Braun, RT Kimball, Rapid and recent diversification of curassows, guans, and chachalacas (Galliformes: Cracidae) out of Mesoamerica: Phylogeny inferred from mitochondrial, intron, and ultraconserved element sequences. *Molecular Phylogenetics and Evolution* **102**, 320–330 (2016).

[69] P Beresford, F Barker, P Ryan, T Crowe, African endemics span the tree of songbirds (Passeri): molecular systematics of several evolutionary ‘enigmas’. *Proceedings of the Royal Society B: Biological Sciences* **272**, 849–858 (2005).

[70] US Johansson, J Fjeldså, RC Bowie, Phylogenetic relationships within Passerida (Aves: Passeriformes): A review and a new molecular phylogeny based on three nuclear intron markers. *Molecular Phylogenetics and Evolution* **48**, 858–876 (2008).

[71] EN Ostrow, TA Catanach, JM Bates, A Aleixo, JD Weckstein, Phylogenomic analysis confirms the relationships among toucans, toucan-barbets, and New World barbets but reveals paraphyly of *Selenidera* toucanets and evidence for mitonuclear discordance. *Ornithology* **140**, ukad022 (2023).

[72] T Cai, et al., Biogeography and diversification of Old World buntings (Aves: Emberizidae): radiation in open habitats. *Journal of Avian Biology* **52**, jav.02672 (2021).

[73] KL Han, MB Robbins, MJ Braun, A multi-gene estimate of phylogeny in the nightjars and nighthawks (Caprimulgidae). *Molecular Phylogenetics and Evolution* **55**, 443–453 (2010).

[74] R Batista, et al., Phylogenomics and biogeography of the world's thrushes (Aves, *Turdus*): new evidence for a more parsimonious evolutionary history. *Proceedings of the Royal Society B: Biological Sciences* **287**, 20192400 (2020).

[75] JP Hruska, et al., Ultraconserved elements resolve the phylogeny and corroborate patterns of molecular rate variation in herons (Aves: Ardeidae). *Ornithology* **140**, ukad005 (2023).

[76] JC Garcia-R, EM Lemmon, AR Lemmon, N French, Phylogenomic Reconstruction Sheds Light on New Relationships and Timescale of Rails (Aves: Rallidae) Evolution. *Diversity* **12**, 70 (2020).

[77] JF Salter, et al., Extensive paraphyly in the typical owl family (Strigidae). *The Auk* **137**, ukz070 (2020).

[78] GC Gibb, et al., New Zealand Passerines Help Clarify the Diversification of Major Songbird Lineages during the Oligocene. *Genome Biology and Evolution* **7**, 2983–2995 (2015).

[79] DW Pietersen, AE McKechnie, R Jansen, IT Little, AD Bastos, Multilocus phylogeny of African pipits and longclaws (Aves: Motacillidae) highlights taxonomic inconsistencies. *Ibis* **161**, 781–792 (2019).

[80] SB Shakya, et al., Observations on the relationships of some Sundaic passerine taxa (Aves: Passeriformes) previously unavailable for molecular phylogenetic study. *Journal of Ornithology* **161**, 651–664 (2020).

[81] MJ Andersen, et al., Ultraconserved elements resolve genus-level relationships in a major Australasian bird radiation (Aves: Meliphagidae). *Emu - Austral Ornithology* **119**, 218–232 (2019).

[82] S Bertelli, AL Porzecanski, Tinamou (tinamidae) Systematics: A Preliminary Combined Analysis of Morphology and Molecules. (2004).

[83] Davies, Owen R, Taxonomy, phylogeny and biogeography of cisticolas (Cisticola spp.) (2015).

[84] US Johansson, et al., A complete multilocus species phylogeny of the tits and chickadees (Aves: Paridae). *Molecular Phylogenetics and Evolution* **69**, 852–860 (2013).

[85] JR Wood, et al., Phylogenetic relationships and terrestrial adaptations of the extinct laughing owl, *Sceloglaux albifacies* (Aves: Strigidae). *Zoological Journal of the Linnean Society* (2016).

[86] JAA Nylander, U Olsson, P Alström, I Sanmartín, Accounting for Phylogenetic Uncertainty in Biogeography: A Bayesian Approach to Dispersal-Vicariance Analysis of the Thrushes (Aves: Turdus). *Systematic Biology* **57**, 257–268 (2008).

[87] U Olsson, M Irestedt, G Sangster, PG Ericson, P Alström, Systematic revision of the avian family Cisticolidae based on a multi-locus phylogeny of all genera. *Molecular Phylogenetics and Evolution* **66**, 790–799 (2013).

[88] TF Wright, et al., A Multilocus Molecular Phylogeny of the Parrots (Psittaciformes): Support for a Gondwanan Origin during the Cretaceous. *Molecular Biology and Evolution* **25**, 2141–2156 (2008).

[89] C Cicero, NA Mason, L Benedict, JD Rising, Behavioral, morphological, and ecological trait evolution in two clades of New World Sparrows (*Aimophila* and *Peucaea* , Passerellidae). *PeerJ* **8**, e9249 (2020).

[90] J Nagy, Z Végvári, Z Varga, Phylogeny, migration and life history: filling the gaps in the origin and biogeography of the Turdus thrushes. *Journal of Ornithology* **160**, 529–543 (2019).

[91] M Stervander, et al., Molecular Species Delimitation of Larks (Aves: Alaudidae), and Integrative Taxonomy of the Genus Calandrella, with the Description of a Range-Restricted African Relic Taxon. *Diversity* **12**, 428 (2020).

[92] M Irestedt, et al., The systematic affinity of the enigmatic Lamprolia victoriae (Aves: Passeriformes)—An example of avian dispersal between New Guinea and Fiji over Miocene intermittent land bridges? *Molecular Phylogenetics and Evolution* **48**, 1218–1222 (2008).

[93] JD Kennedy, et al., Diversification and community assembly of the world's largest tropical island. *Global Ecology and Biogeography* **31**, 1078–1089 (2022).

[94] NI Mann, FK Barker, JA Graves, KA Dingess-Mann, PJ Slater, Molecular data delineate four genera of "Thryothorus" wrens. *Molecular Phylogenetics and Evolution* **40**, 750–759 (2006).

[95] JF Salter, et al., Historical specimens and the limits of subspecies phylogenomics in the New World quails (Odontophoridae). *Molecular Phylogenetics and Evolution* **175**, 107559 (2022).

[96] RJ Den Tex, JA Leonard, A molecular phylogeny of Asian barbets: Speciation and extinction in the tropics. *Molecular Phylogenetics and Evolution* **68**, 1–13 (2013).

[97] J Fuchs, JI Ohlson, PGP Ericson, E Pasquet, Synchronous intercontinental splits between assemblages of woodpeckers suggested by molecular data. *Zoologica Scripta* **36**, 11–25 (2007).

[98] L Joseph, A Toon, EE Schirtzinger, TF Wright, Molecular systematics of two enigmatic genera Psittacella and Pezoporus illuminate the ecological radiation of Australo-Papuan parrots (Aves: Psittaciformes). *Molecular Phylogenetics and Evolution* **59**, 675–684 (2011).

[99] T Arbabi, J Gonzalez, M Wink, A re-evaluation of phylogenetic relationships within reed warblers (Aves: Acrocephalidae) based on eight molecular loci and ISSR profiles. *Molecular Phylogenetics and Evolution* **78**, 304–313 (2014).

[100] BW Benz, MB Robbins, Molecular phylogenetics, vocalizations, and species limits in Celeus woodpeckers (Aves: Picidae). *Molecular Phylogenetics and Evolution* **61**, 29–44 (2011).

[101] GC Gibb, LD Shepherd, Recent evolution of extreme sexual dimorphism in the huia (Heteralocha acutirostris; Callaeidae). *Molecular Phylogenetics and Evolution* **175**, 107575 (2022).

[102] TS Imfeld, FK Barker, RT Brumfield, Mitochondrial genomes and thousands of ultraconserved elements resolve the taxonomy and historical biogeography of the Euphonia and Chlorophonia finches (Passeriformes: Fringillidae). *The Auk* **137**, ukaa016 (2020).

[103] H Lerner, M Meyer, H James, M Hofreiter, R Fleischer, Multilocus Resolution of Phylogeny and Timescale in the Extant Adaptive Radiation of Hawaiian Honeycreepers. *Current Biology* **21**, 1838–1844 (2011).

[104] IJ Lovette, et al., Phylogenetic relationships of the mockingbirds and thrashers (Aves: Mimidae). *Molecular Phylogenetics and Evolution* **63**, 219–229 (2012).

[105] ES Bridge, AW Jones, AJ Baker, A phylogenetic framework for the terns (Sternini) inferred from mtDNA sequences: implications for taxonomy and plumage evolution. *Molecular Phylogenetics and Evolution* **35**, 459–469 (2005).

[106] RG Moyle, Phylogenetics of barbets (Aves: Piciformes) based on nuclear and mitochondrial DNA sequence data. *Molecular Phylogenetics and Evolution* **30**, 187–200 (2004).

[107] FE Rheindt, JA Norman, L Christidis, Phylogenetic relationships of tyrant-flycatchers (Aves: Tyrannidae), with an emphasis on the elaeniine assemblage. *Molecular Phylogenetics and Evolution* **46**, 88–101 (2008).

[108] A Cibois, J Thibault, E Pasquet, Systematics of the extinct reed warblers *Acrocephalus* of the Society Islands of eastern Polynesia. *Ibis* **150**, 365–376 (2008).

[109] DM Hooper, U Olsson, P Alström, The Rusty-tailed Flycatcher (Muscicapa ruficauda; Aves: Muscicapidae) is a member of the genus Ficedula. *Molecular Phylogenetics and Evolution* **102**, 56–61 (2016).

[110] RG Moyle, A Molecular Phylogeny of Kingfishers (Alcedinidae) With Insights Into Early Biogeographic History. *The Auk* **123**, 487–499 (2006).

[111] JI Ohlson, J Fjeldså, PG Ericson, Molecular phylogeny of the manakins (Aves: Passeriformes: Pipridae), with a new classification and the description of a new genus. *Molecular Phylogenetics and Evolution* **69**, 796–804 (2013).

[112] JF Ornelas, C González, A Espinosa De Los Monteros, Uncorrelated evolution between vocal and plumage coloration traits in the trogons: a comparative study. *Journal of Evolutionary Biology* **22**, 471–484 (2009).

[113] RG Moyle, BD Marks, Phylogenetic relationships of the bulbuls (Aves: Pycnonotidae) based on mitochondrial and nuclear DNA sequence data. *Molecular Phylogenetics and Evolution* **40**, 687–695 (2006).

[114] S Treplin, et al., Molecular phylogeny of songbirds (Aves: Passeriformes) and the relative utility of common nuclear marker loci. *Cladistics* **24**, 328–349 (2008).

[115] PGP Ericson, et al., Parallel Evolution of Bower-Building Behavior in Two Groups of Bowerbirds Suggested by Phylogenomics. *Systematic Biology* **69**, 820–829 (2020).

[116] M Schweizer, TF Wright, JV Peñalba, EE Schirtzinger, L Joseph, Molecular phylogenetics suggests a New Guinean origin and frequent episodes of founder-event speciation in the nectarivorous lories and lorikeets (Aves: Psittaciformes). *Molecular Phylogenetics and Evolution* **90**, 34–48 (2015).

[117] M Päckert, J Martens, M Wink, A Feigl, DT Tietze, Molecular phylogeny of Old World swifts (Aves: Apodiformes, Apodidae, Apus and Tachymarptis) based on mitochondrial and nuclear markers. *Molecular Phylogenetics and Evolution* **63**, 606–616 (2012).

[118] N Dos Remedios, PL Lee, T Burke, T Székely, C Küpper, North or south? Phylogenetic and biogeographic origins of a globally distributed avian clade. *Molecular Phylogenetics and Evolution* **89**, 151–159 (2015).

[119] KA Jønsson, et al., Phylogeny and biogeography of Oriolidae (Aves: Passeriformes). *Ecography* **33**, 232–241 (2010).

[120] A Ödeen, O Håstad, P Alström, Evolution of ultraviolet vision in the largest avian radiation - the passerines. *BMC Evolutionary Biology* **11**, 313 (2011).

[121] SL Pereira, KP Johnson, DH Clayton, AJ Baker, Mitochondrial and Nuclear DNA Sequences Support a Cretaceous Origin of Columbiformes and a Dispersal-Driven Radiation in the Paleogene. *Systematic Biology* **56**, 656–672 (2007).

[122] C Pitra, D Lieckfeldt, S Frahnert, J Fickel, Phylogenetic Relationships and Ancestral Areas of the Bustards (Gruiformes: Otididae), Inferred from Mitochondrial DNA and Nuclear Intron Sequences. *Molecular Phylogenetics and Evolution* **23**, 63–74 (2002).

[123] D Zuccon, A Cibois, E Pasquet, PG Ericson, Nuclear and mitochondrial sequence data reveal the major lineages of starlings, mynas and related taxa. *Molecular Phylogenetics and Evolution* **41**, 333–344 (2006).

[124] P Alström, S Höhna, M Gelang, PG Ericson, U Olsson, Non-monophyly and intricate morphological evolution within the avian family Cettiidae revealed by multilocus analysis of a taxonomically densely sampled dataset. *BMC Evolutionary Biology* **11**, 352 (2011).

[125] P Alström, et al., Dramatic niche shifts and morphological change in two insular bird species. *Royal Society Open Science* **2**, 140364 (2015).

[126] R Moyle, R.G., J Fuchs, E Pasquet, B D. Marks, Feeding behavior, toe count, and the phylogenetic relationships among alcedinine kingfishers (Alcedininae). *Journal of Avian Biology* **38**, 317–326 (2007).

[127] Nyári, BW Benz, KA Jønsson, J Fjeldså, RG Moyle, Phylogenetic relationships of fantails (Aves: Rhipiduridae). *Zoologica Scripta* **38**, 553–561 (2009).

[128] PG Ericson, S Klopfstein, M Irestedt, JM Nguyen, JA Nylander, Dating the diversification of the major lineages of Passeriformes (Aves). *BMC Evolutionary Biology* **14**, 8 (2014).

[129] E Pasquet, et al., Evolution within the nuthatches (Sittidae: Aves, Passeriformes): molecular phylogeny, biogeography, and ecological perspectives. *Journal of Ornithology* **155**, 755–765 (2014).

[130] SL Pereira, AJ Baker, DNA evidence for a Paleocene origin of the Alcidae (Aves: Charadriiformes) in the Pacific and multiple dispersals across northern oceans. *Molecular Phylogenetics and Evolution* **46**, 430–445 (2008).

[131] MA Russello, G Amato, A molecular phylogeny of Amazona: implications for Neotropical parrot biogeography, taxonomy, and conservation. *Molecular Phylogenetics and Evolution* **30**, 421–437 (2004).

[132] DL Slager, C Battey, RW Bryson, G Voelker, J Klicka, A multilocus phylogeny of a major New World avian radiation: The Vireonidae. *Molecular Phylogenetics and Evolution* **80**, 95–104 (2014).

[133] J Fuchs, et al., Tracing the colonization history of the Indian Ocean scops-owls (Strigiformes: Otus) with further insight into the spatio-temporal origin of the Malagasy avifauna. *BMC Evolutionary Biology* **8**, 197 (2008).

[134] RB Harris, MD Carling, IJ Lovette, The Influence of Sampling Design on Species Tree Inference: A New Relationship for the New World Chickadees (aves: poecile): Species Tree Inference in Chickadees. *Evolution* **68**, 501–513 (2014).

[135] JY Lee, L Joseph, SV Edwards, A Species Tree for the Australo-Papuan Fairy-wrens and Allies (Aves: Maluridae). *Systematic Biology* **61**, 253 (2012).

[136] BD Marks, JD Weckstein, RG Moyle, Molecular phylogenetics of the bee-eaters (Aves: Meropidae) based on nuclear and mitochondrial DNA sequence data. *Molecular Phylogenetics and Evolution* **45**, 23–32 (2007).

[137] RG Moyle, Phylogeny and biogeographical history of Trogoniformes, a pantropical bird order. *Biological Journal of the Linnean Society* **84**, 725–738 (2005).

[138] DT Tietze, M Päckert, J Martens, H Lehmann, YH Sun, Complete phylogeny and historical biogeography of true rosefinches (Aves: *Carpodacus*): Rosefinch Phylogeny and Historical Biogeography. *Zoological Journal of the Linnean Society* **169**, 215–234 (2013).

[139] RT Brumfield, SV Edwards, Evolution into and Out of the Andes: A Bayesian Analysis of Historical Diversification in thamnophilus Antshrikes: Evolution into and Out of the Andes. *Evolution* **61**, 346–367 (2007).

[140] SM Dantas, et al., Molecular systematics of the new world screech-owls (Megascops: Aves, Strigidae): biogeographic and taxonomic implications. *Molecular Phylogenetics and Evolution* **94**, 626–634 (2016).

[141] KY Njabo, MD Sorenson, Origin of Bannerman's Turaco *Tauraco bannermani* in relation to historical climate change and the distribution of West African montane forests. *Ostrich* **80**, 1–7 (2009).

[142] RW Bryson, BC Faircloth, WLE Tsai, JE McCormack, J Klicka, Target enrichment of thousands of ultraconserved elements sheds new light on early relationships within New World sparrows (Aves: Passerellidae). *The Auk* **133**, 451–458 (2016).

[143] IJ Lovette, BV McCleery, AL Talaba, DR Rubenstein, A complete species-level molecular phylogeny for the "Eurasian" starlings (Sturnidae: Sturnus, Acridotheres, and allies): Recent diversification in a highly social and dispersive avian group. *Molecular Phylogenetics and Evolution* **47**, 251–260 (2008).

[144] BT Smith, et al., Species delimitation and biogeography of the gnatcatchers and gnatwrens (Aves: Polioptilidae). *Molecular Phylogenetics and Evolution* **126**, 45–57 (2018).

[145] ND White, MJ Braun, Extracting phylogenetic signal from phylogenomic data: Higher-level relationships of the nightbirds (Strisores). *Molecular Phylogenetics and Evolution* **141**, 106611 (2019).

[146] N Buainain, et al., Biogeography of a neotropical songbird radiation reveals similar diversification dynamics between montane and lowland clades. *Journal of Biogeography* **49**, 1260–1273 (2022).

[147] RG Moyle, RT Chesser, RO Prum, P Schikler, J Cracraft, Phylogeny and Evolutionary History of Old World Suboscine Birds (Aves: Eurylaimides). *American Museum Novitates* **3544**, 1 (2006).

[148] J Ottenburghs, et al., A tree of geese: A phylogenomic perspective on the evolutionary history of True Geese. *Molecular Phylogenetics and Evolution* **101**, 303–313 (2016).

[149] G Besnard, et al., Valuing museum specimens: high-throughput DNA sequencing on historical collections of New Guinea crowned pigeons (*Goura*): Valuing historical specimens with museomics. *Biological Journal of the Linnean Society* **117**, 71–82 (2016).

[150] LC Campillo, CH Oliveros, FH Sheldon, RG Moyle, Genomic data resolve gene tree discordance in spiderhunters (Nectariniidae, Arachnothera). *Molecular Phylogenetics and Evolution* **120**, 151–157 (2018).

[151] J Fuchs, JA Johnson, DP Mindell, Molecular systematics of the caracaras and allies (Falconidae: Polyborinae) inferred from mitochondrial and nuclear sequence data. *Ibis* **154**, 520–532 (2012).

[152] A Gavryushkina, et al., Bayesian Total-Evidence Dating Reveals the Recent Crown Radiation of Penguins. *Systematic Biology* p. syw060 (2016).

[153] X Zhou, C Yao, Q Lin, W Fang, X Chen, Complete mitochondrial genomes render the Night Heron genus Gorsachius non-monophyletic. *Journal of Ornithology* **157**, 505–513 (2016).

[154] C Donne-Goussé, V Laudet, C Hänni, A molecular phylogeny of anseriformes based on mitochondrial DNA analysis. *Molecular Phylogenetics and Evolution* **23**, 339–356 (2002).

[155] J Gonzalez, M Wink, Phylogenetic position of the monotypic Des Murs' Wiretail (Sylviorthorhynchus desmursii, Aves: Furnariidae) based on mitochondrial and nuclear DNA. *Journal of Ornithology* **149**, 393–398 (2008).

[156] C Krajewski, JT Sipiorski, FE Anderson, Complete Mitochondrial Genome Sequences and the Phylogeny of Cranes (Gruiformes: Gruidae). *The Auk* **127**, 440–452 (2010).

[157] N Nováková, J Robovský, Behaviour of cranes (family Gruidae) mirrors their phylogenetic relationships. *Avian Research* **12**, 40 (2021).

[158] B Slikas, Phylogeny of the Avian Family Ciconiidae (Storks) Based on CytochromebSequences and DNA–DNA Hybridization Distances. *Molecular Phylogenetics and Evolution* **8**, 275–300 (1997).

[159] JA Vianna, et al., Genome-wide analyses reveal drivers of penguin diversification. *Proceedings of the National Academy of Sciences* **117**, 22303–22310 (2020).

[160] RW Bryson, et al., Diversification across the New World within the 'blue' cardinalids (Aves: Cardinalidae). *Journal of Biogeography* **41**, 587–599 (2014).

[161] GK Chambers, C Moeke, R Steel, J Trueman, Phylogenetic analysis of the 24 named albatross taxa based on full mitochondrial cytochrome b DNA sequences. *Notornis* (2009).

[162] R Dor, RJ Safran, FH Sheldon, DW Winkler, IJ Lovette, Phylogeny of the genus Hirundo and the Barn Swallow subspecies complex. *Molecular Phylogenetics and Evolution* **56**, 409–418 (2010).

[163] MG Fain, C Krajewski, P Houde, Phylogeny of "core Gruiformes" (Aves: Grues) and resolution of the Limpkin–Sungrebe problem. *Molecular Phylogenetics and Evolution* **43**, 515–529 (2007).

[164] M Irestedt, KA Jønsson, J Fjeldså, L Christidis, PG Ericson, An unexpectedly long history of sexual selection in birds-of-paradise. *BMC Evolutionary Biology* **9**, 235 (2009).

[165] DT Ksepka, RE Fordyce, T Ando, CM Jones, New fossil penguins (Aves, Sphenisciformes) from the Oligocene of New Zealand reveal the skeletal plan of stem penguins. *Journal of Vertebrate Paleontology* **32**, 235–254 (2012).

[166] E Quintero, CC Ribas, J Cracraft, The Andean *Hapalopsittaca* parrots (Psittacidae, Aves): an example of montane-tropical lowland vicariance. *Zoologica Scripta* **42**, 28–43 (2013).

[167] G Voelker, Molecular phylogenetics and the historical biogeography of dippers (*Cinclus*). *Ibis* **144**, 577–584 (2002).

[168] M Moltesen, M Irestedt, J Fjeldså, PG Ericson, KA Jønsson, Molecular phylogeny of Chloropseidae and Irenidae – Cryptic species and biogeography. *Molecular Phylogenetics and Evolution* **65**, 903–914 (2012).

[169] AD Sweet, KP Johnson, Patterns of diversification in small New World ground doves are consistent with major geologic events. *The Auk* **132**, 300–312 (2015).

[170] JA Chaves, JR Hidalgo, J Klicka, Biogeography and evolutionary history of the neotropical genus saltator (aves: Thraupini). *Journal of Biogeography* **40**, 2180–2190 (2013).

[171] J Fuchs, S Chen, JA Johnson, DP Mindell, Pliocene diversification within the South American Forest falcons (Falconidae: Micrastur). *Molecular Phylogenetics and Evolution* **60**, 398–407 (2011).

[172] JC García–R, GC Gibb, SA Trewick, Eocene Diversification of Crown Group Rails (Aves: Gruiformes: Rallidae). *PLoS ONE* **9**, e109635 (2014).

[173] US Johansson, M Irestedt, Y Qu, PG Ericson, Phylogenetic relationships of rollers (Coraciidae) based on complete mitochondrial genomes and fifteen nuclear genes. *Molecular Phylogenetics and Evolution* **126**, 17–22 (2018).

[174] HRL Lerner, MC Klaver, DP Mindell, Molecular Phylogenetics of the Buteonine Birds of Prey (accipitridae). *The Auk* **125**, 304–315 (2008).

[175] JM McCullough, et al., Wallacean and Melanesian Islands Promote Higher Rates of Diversification within the Global Passerine Radiation Corvides. *Systematic Biology* **71**, 1423–1439 (2022).

[176] RG Moyle, J Cracraft, M Lakim, J Nais, FH Sheldon, Reconsideration of the phylogenetic relationships of the enigmatic Bornean Bristlehead (Pityriasis gymnocephala). *Molecular Phylogenetics and Evolution* **39**, 893–898 (2006).

[177] G Oatley, RE Simmons, J Fuchs, A molecular phylogeny of the harriers (Circus, Accipitridae) indicate the role of long distance dispersal and

migration in diversification. *Molecular Phylogenetics and Evolution* **85**, 150–160 (2015).

[178] J Ramirez, C Miyaki, S Del Lama, Molecular phylogeny of Threskiornithidae (Aves: Pelecaniformes) based on nuclear and mitochondrial DNA. *Genetics and Molecular Research* **12**, 2740–2750 (2013).

[179] J Younger, et al., *Diversification of a cryptic radiation, a closer look at Madagascar's recently recognized bird family.* (2019).

[180] JC Garcia-R., SA Trewick, Dispersal and speciation in purple swamphens (Rallidae: *Porphyrio*). *The Auk* **132**, 140–155 (2015).

[181] O Johnson, JT Howard, RT Brumfield, Systematics of a Neotropical clade of dead-leaf-foraging antwrens (Aves: Thamnophilidae; Epinecrophylla). *Molecular Phylogenetics and Evolution* **154**, 106962 (2021).

[182] B Liu, et al., Explosive radiation and spatial expansion across the cold environments of the Old World in an avian family. *Ecology and Evolution* **7**, 6346–6357 (2017).

[183] M Päckert, et al., Horizontal and elevational phylogeographic patterns of Himalayan and Southeast Asian forest passerines (Aves: Passeriformes). *Journal of Biogeography* **39**, 556–573 (2012).

[184] S Patel, JD Weckstein, JS Patané, JM Bates, A Aleixo, Temporal and spatial diversification of Pteroglossus araçaris (AVES: Ramphastidae) in the neotropics: Constant rate of diversification does not support an increase in radiation during the Pleistocene. *Molecular Phylogenetics and Evolution* **58**, 105–115 (2011).

[185] S Patterson, J Morris-Pocock, V Friesen, A multilocus phylogeny of the Sulidae (Aves: Pelecaniformes). *Molecular Phylogenetics and Evolution* **58**, 181–191 (2011).

[186] SL Pereira, A Wajntal, The historical biogeography of Pteroglossus aracaris (Aves, Piciformes, Ramphastidae) based on Bayesian analysis of mitochondrial DNA sequences. *Genetics and Molecular Biology* **31**, 964–973 (2008).

[187] SL Pereira, AJ Baker, Vicariant Speciation of Curassows (Aves, Cracidae): A Hypothesis Based on Mitochondrial DNA Phylogeny. *The Auk* **121**, 682–694 (2004).

[188] NE White, et al., The evolutionary history of cockatoos (Aves: Psittaciformes: Cacatuidae). *Molecular Phylogenetics and Evolution* **59**, 615–622 (2011).

[189] T Yonezawa, et al., Phylogenomics and Morphology of Extinct Paleognaths Reveal the Origin and Evolution of the Ratites. *Current Biology* **27**, 68–77 (2017).

[190] Z Zhang, et al., Unexpected divergence and lack of divergence revealed in continental Asian Cyornis flycatchers (Aves: Muscicapidae). *Molecular Phylogenetics and Evolution* **94**, 232–241 (2016).

[191] BW Benz, MB Robbins, AT Peterson, Evolutionary history of woodpeckers and allies (Aves: Picidae): Placing key taxa on the phylogenetic tree. *Molecular Phylogenetics and Evolution* **40**, 389–399 (2006).

[192] DJ Cerasale, R Dor, DW Winkler, IJ Lovette, Phylogeny of the Tachycineta genus of New World swallows: Insights from complete mitochondrial genomes. *Molecular Phylogenetics and Evolution* **63**, 64–71 (2012).

[193] S Lamichhaney, et al., Evolution of Darwin's finches and their beaks revealed by genome sequencing. *Nature* **518**, 371–375 (2015).

[194] HL Lutz, JD Weckstein, JS Patané, JM Bates, A Aleixo, Biogeography and spatio-temporal diversification of Selenidera and Andigena Tou-

cans (Aves: Ramphastidae). *Molecular Phylogenetics and Evolution* **69**, 873–883 (2013).

[195] WS Moore, LC Overton, KJ Miglia, Mitochondrial DNA based phylogeny of the woodpecker genera Colaptes and Piculus, and implications for the history of woodpecker diversification in South America. *Molecular Phylogenetics and Evolution* **58**, 76–84 (2011).

[196] CC Ribas, CY Miyaki, J Cracraft, Phylogenetic relationships, diversification and biogeography in Neotropical *Brotogeris* parakeets. *Journal of Biogeography* **36**, 1712–1729 (2009).

[197] NH Rice, Phylogenetic Relationships of Antpitta Genera (Passeriformes: Formicariidae). *The Auk* **122**, 673–683 (2005).

[198] G Spellman, A Cibois, R Moyle, K Winker, F Keithbarker, Clarifying the systematics of an enigmatic avian lineage: What is a bombycillid? *Molecular Phylogenetics and Evolution* **49**, 1036–1040 (2008).

[199] RM Zink, RC Blackwell, Molecular Systematics and Biogeography of Aridland Gnatcatchers (GenusPolioptila) and Evidence Supporting Species Status of the California Gnatcatcher (Polioptila californica). *Molecular Phylogenetics and Evolution* **9**, 26–32 (1998).

[200] F Zino, R Brown, M Biscoito, The separation of *Pterodroma madeira* (Zino's Petrel) from *Pterodroma feae* (Fea's Petrel) (Aves: Procellariidae). *Ibis* **150**, 326–334 (2008).

[201] M Aliabadian, M Kaboli, R Prodon, V Nijman, M Vences, Phylogeny of Palaearctic wheatears (genus Oenanthe)—Congruence between morphometric and molecular data. *Molecular Phylogenetics and Evolution* **42**, 665–675 (2007).

[202] A Cibois, et al., Phylogeny and biogeography of the fruit doves (Aves: Columbidae). *Molecular Phylogenetics and Evolution* **70**, 442–453 (2014).

[203] M Kennedy, SA Taylor, P Nádvorník, HG Spencer, The phylogenetic relationships of the extant pelicans inferred from DNA sequence data. *Molecular Phylogenetics and Evolution* **66**, 215–222 (2013).

[204] RG Moyle, B Slikas, LA Whittingham, DW Winkler, FH Sheldon, DNA Sequence assessment of phylogenetic relationships among New World martins (Hirundinidae: Progne). *The Wilson Journal of Ornithology* **120**, 683–691 (2008).

[205] B Nguembock, J Fjeldså, C Cruaud, E Pasquet, Molecular phylogenetic analysis of all members of the genus *Elminia* confirms their presence within the Stenostiridae clade. *Zoologica Scripta* **37**, 591–602 (2008).

[206] SL Pereira, AJ Baker, A Wajntal, Combined Nuclear and Mitochondrial DNA Sequences Resolve Generic Relationships within the Cracidae (Galliformes, Aves). *Systematic Biology* **51**, 946–958 (2002).

[207] CC Ribas, L Joseph, CY Miyaki, Molecular Systematics and Patterns of Diversification in Pyrrhura (Psittacidae), with Special Reference to the Picta-Leucotis Complex. *The Auk* **123**, 660–680 (2006).

[208] M Schweizer, M Güntert, ST Hertwig, Phylogeny and biogeography of the parrot genus *Prioniturus* (Aves: Psittaciformes). *Journal of Zoological Systematics and Evolutionary Research* **50**, 145–156 (2012).

[209] Blechschmidt, K., et al., Investigation on the molecular systematics of skuas (Stercorariidae). *Zoologische JahrbiXcher Systematik,* **120**, 379–387. (1993).

[210] RB Harris, SM Birks, AD Leaché, Incubator birds: biogeographical origins and evolution of underground nesting in megapodes (Galliformes: Megapodiidae). *Journal of Biogeography* **41**, 2045–2056 (2014).

[211] JA Johnson, JW Brown, J Fuchs, DP Mindell, Multi-locus phylogenetic inference among New World Vultures (Aves: Cathartidae). *Molecular Phylogenetics and Evolution* **105**, 193–199 (2016).

[212] AE Moncrieff, BC Faircloth, RT Brumfield, Systematics of Lepidothrix manakins (Aves: Passeriformes: Pipridae) using RADcap markers. *Molecular Phylogenetics and Evolution* **173**, 107525 (2022).

[213] M Schweizer, H Shirihai, Phylogeny of the Oenanthe lugens complex (Aves, Muscicapidae: Saxicolinae): Paraphyly of a morphologically cohesive group within a recent radiation of open-habitat chats. *Molecular Phylogenetics and Evolution* **69**, 450–461 (2013).

[214] BT Smith, CC Ribas, BM Whitney, BE HernÁndez-baÑos, J Klicka, Identifying biases at different spatial and temporal scales of diversification: a case study in the Neotropical parrotlet genus *Forpus*. *Molecular Ecology* **22**, 483–494 (2013).

[215] S Subramanian, G Beans-Picón, SK Swaminathan, CD Millar, DM Lambert, Evidence for a recent origin of penguins. *Biology Letters* **9**, 20130748 (2013).

[216] P Alström, et al., Multiple species within the Striated Prinia *Prinia crinigera*– Brown Prinia *P. polychroa* complex revealed through an integrative taxonomic approach. *Ibis* **162**, 936–967 (2020).

[217] P Alström, et al., Taxonomy of the White-browed Shortwing (Brachypteryx montana) complex on mainland Asia and Taiwan: an integrative approach supports recognition of three instead of one species. *Avian Research* **9**, 34 (2018).

[218] JK Armenta, JD Weckstein, DF Lane, Geographic Variation in Mitochondrial DNA Sequences of an Amazonian Nonpasserine: The Black-Spotted Barbet Complex. *The Condor* **107**, 527–536 (2005).

[219] E Bonaccorso, JM Guayasamin, AT Peterson, AG Navarro-Sigüenza, Molecular phylogeny and systematics of Neotropical toucanets in the genus *Aulacorhynchus* (Aves, Ramphastidae). *Zoologica Scripta* **40**, 336–349 (2011).

[220] J Bruxaux, et al., Recovering the evolutionary history of crowned pigeons (Columbidae: Goura): Implications for the biogeography and conservation of New Guinean lowland birds. *Molecular Phylogenetics and Evolution* **120**, 248–258 (2018).

[221] RT Chesser, CKL Yeung, CT Yao, XH Tian, SH Li, Molecular phylogeny of the spoonbills (Aves: Threskiornithidae) based on mitochondrial DNA. (2010).

[222] J Dumbacher, K Deiner, L Thompson, R Fleischer, Phylogeny of the avian genus Pitohui and the evolution of toxicity in birds. *Molecular Phylogenetics and Evolution* **49**, 774–781 (2008).

[223] J Dumbacher, Phylogeny of the owlet-nightjars (Aves: Aegothelidae) based on mitochondrial DNA sequence. *Molecular Phylogenetics and Evolution* **29**, 540–549 (2003).

[224] B Milá, et al., A new, undescribed species of *Melanocharis* berrypecker from western New Guinea and the evolutionary history of the family Melanocharitidae. *Ibis* **163**, 1310–1329 (2021).

[225] KJ Mitchell, et al., Ancient DNA reveals elephant birds and kiwi are sister taxa and clarifies ratite bird evolution. *Science* **344**, 898–900 (2014).

[226] M Päckert, J Martens, LL Severinghaus, The Taiwan Firecrest (Regulus goodfellowi) belongs to the Goldcrest assemblage (Regulus regulus s. l.): evidence from mitochondrial DNA and the territorial song of the Regulidae. *Journal of Ornithology* **150**, 205–220 (2009).

[227] JS Patané, JD Weckstein, A Aleixo, JM Bates, Evolutionary history of Ramphastos toucans: Molecular phylogenetics, temporal diversification, and biogeography. *Molecular Phylogenetics and Evolution* **53**, 923–934 (2009).

[228] A Shipham, DJ Schmidt, L Joseph, JM Hughes, Phylogenetic analysis of the Australian rosella parrots (Platycercus) reveals discordance among molecules and plumage. *Molecular Phylogenetics and Evolution* **91**, 150–159 (2015).

[229] CR Torres, LM Ogawa, MA Gillingham, B Ferrari, M Van Tuinen, A multi-locus inference of the evolutionary diversification of extant flamingos (Phoenicopteridae). *BMC Evolutionary Biology* **14**, 36 (2014).

[230] ND White, C Mitter, MJ Braun, Ultraconserved elements resolve the phylogeny of potoos (Aves: Nyctibiidae). *Journal of Avian Biology* **48**, 872–880 (2017).

[231] LA Whittingham, FH Sheldon, ST Emlen, Molecular Phylogeny of Jacanas and its Implications for Morphologic and Biogeographic Evolution. *The Auk* **117**, 22–32 (2000).

[232] P Alström, et al., Integrative taxonomy of the Plain-backed Thrush (Zoothera mollissima) complex (Aves, Turdidae) reveals cryptic species, including a new species. *Avian Research* **7**, 1 (2016).

[233] D Boertmann, Phylogeny of the divers, family Gaviidae (Aves). *Steenstrupia* **16**, 21–36 (1990).

[234] JC Illera, DS Richardson, B Helm, JC Atienza, BC Emerson, Phylogenetic relationships, biogeography and speciation in the avian genus Saxicola. *Molecular Phylogenetics and Evolution* **48**, 1145–1154 (2008).

[235] A Manegold, Composition and phylogenetic affinities of vangas (Vangidae, Oscines, Passeriformes) based on morphological characters. *Journal of Zoological Systematics and Evolutionary Research* **46**, 267–277 (2008).

[236] M Melo, J Fuchs, Phylogenetic relationships of the Gulf of Guinea *Alcedo* kingfishers. *Ibis* **150**, 633–639 (2008).

[237] LM Ogawa, PC Pulgarin, DA Vance, J Fjeldså, M Van Tuinen, Opposing demographic histories reveal rapid evolution in grebes (Aves: Podicipedidae). *The Auk* **132**, 771–786 (2015).

[238] PC Pulgarín-R, BT Smith, RW Bryson, GM Spellman, J Klicka, Multilocus phylogeny and biogeography of the New World Pheucticus grosbeaks (Aves: Cardinalidae). *Molecular Phylogenetics and Evolution* **69**, 1222–1227 (2013).

[239] Q Sprengelmeyer, A Phylogenetic Reevaluation of the Genus Gavia (aves: Gaviiformes) Using Next-Generation Sequencing. *All NMU Master's Theses* (2014).

[240] G Voelker, JE Light, Palaeoclimatic events, dispersal and migratory losses along the Afro-European axis as drivers of biogeographic distribution in Sylvia warblers. *BMC Evolutionary Biology* **11**, 163 (2011).

[241] PB Zwiers, G Borgia, RC Fleischer, Plumage based classification of the bowerbird genus Sericulus evaluated using a multi-gene, multi-genome analysis. *Molecular Phylogenetics and Evolution* **46**, 923–931 (2008).

[242] M Arshad, J Gonzalez, AA El-Sayed, T Osborne, M Wink, Phylogeny and phylogeography of critically endangered Gyps species based on nuclear and mitochondrial markers. *Journal of Ornithology* **150**, 419–430 (2009).

[243] KP Johnson, JD Weckstein, S Virrueta Herrera, J Doña, The interplay between host biogeography and phylogeny in structuring diversification of the feather louse genus Penenirmus. *Molecular Phylogenetics and Evolution* **165**, 107297 (2021).

[244] M Kennedy, SS Seneviratne, UK Mendis, HG Spencer, Sorting out the Snakebirds: The species status, phylogeny, and biogeography of the Darters (Aves: Anhingidae). *Journal of Zoological Systematics and Evolutionary Research* **57**, 892–899 (2019).

[245] M Lecroy, F Barker, A New Species of Bush-warbler from Bougainville Island and a Monophyletic Origin for Southwest Pacific Cettia. *American Museum Novitates* **3511**, 1 (2006).

[246] KJ Mitchell, et al., Ancient mitochondrial genomes clarify the evolutionary history of New Zealand's enigmatic acanthisittid wrens. *Molecular Phylogenetics and Evolution* **102**, 295–304 (2016).

[247] S Reddy, et al., Why Do Phylogenomic Data Sets Yield Conflicting Trees? Data Type Influences the Avian Tree of Life more than Taxon Sampling. *Systematic Biology* **66** (2017).

[248] ES Tavares, AJ Baker, SL Pereira, CY Miyaki, Phylogenetic Relationships and Historical Biogeography of Neotropical Parrots (Psittaciformes: Psittacidae: Arini) Inferred from Mitochondrial and Nuclear DNA Sequences. *Systematic Biology* **55**, 454–470 (2006).

[249] RM Zink, DL Dittmann, J Klicka, RC Blackwell-Rago, Evolutionary Patterns of Morphometrics, Allozymes, and Mitochondrial DNA in Thrashers (Genus Toxostoma). *The Auk* **116**, 1021–1038 (1999).

[250] P Alström, et al., Morphology, vocalizations, and mitochondrial DNA suggest that the Graceful Prinia is two species. *Ornithology* **138**, ukab014 (2021).

[251] A Corl, H Ellegren, Sampling strategies for species trees: The effects on phylogenetic inference of the number of genes, number of individuals, and whether loci are mitochondrial, sex-linked, or autosomal. *Molecular Phylogenetics and Evolution* **67**, 358–366 (2013).

[252] J Feinstein, X Yang, S Li, Molecular systematics and historical biogeography of the Black-browed Barbet species complex (*Megalaima oorti*). *Ibis* **150**, 40–49 (2008).

[253] CC Ribas, A Aleixo, ACR Nogueira, CY Miyaki, J Cracraft, A palaeobiogeographic model for biotic diversification within Amazonia over the

past three million years. *Proceedings of the Royal Society B: Biological Sciences* **279**, 681–689 (2012).

[254] F Woog, M Wink, E Rastegar-Pouyani, J Gonzalez, B Helm, Distinct taxonomic position of the Madagascar stonechat (Saxicola torquatus sibilla) revealed by nucleotide sequences of mitochondrial DNA. *Journal of Ornithology* **149**, 423–430 (2008).

[255] M Aggerbeck, J Fjeldså, L Christidis, PH Fabre, KA Jønsson, Resolving deep lineage divergences in core corvoid passerine birds supports a proto-Papuan island origin. *Molecular Phylogenetics and Evolution* **70**, 272–285 (2014).

[256] P Alström, et al., Multiple species delimitation approaches applied to the avian lark genus *Alaudala*. *Molecular Phylogenetics and Evolution* **154**, 106994 (2021).

[257] AJ Baker, SL Pereira, TA Paton, Phylogenetic relationships and divergence times of Charadriiformes genera: multigene evidence for the Cretaceous origin of at least 14 clades of shorebirds. *Biology Letters* **3**, 205–210 (2007).

[258] GM García-Deras, et al., Phylogenetic relationships within the genus Cynanthus (Aves: Trochilidae), with emphasis on C. doubledayi. (2008).

[259] KA Jønsson, et al., Molecular phylogenetics and diversification within one of the most geographically variable bird species complexes Pachycephala pectoralis/melanura. *Journal of Avian Biology* **39**, 473–478 (2008).

[260] LC Overton, DD Rhoads, Molecular phylogenetic relationships based on mitochondrial and nuclear gene sequences for the Todies (*Todus*, Todidae) of the Caribbean. *Molecular Phylogenetics and Evolution* **32**, 524–538 (2004).

[261] TL Parchman, CW Benkman, ET Mezquida, Coevolution Between Hispaniolan Crossbills and Pine: Does More Time Allow for Greater Phenotypic Escalation at Lower Latitude? *Evolution* **61**, 2142–2153 (2007).

[262] E Scholes III, Evolution of the courtship phenotype in the bird of paradise genus Parotia (Aves: Paradisaeidae): homology, phylogeny, and modularity. *Biological Journal of the Linnean Society* **94**, 491–504 (2008).
